# Nanogalvanic cell catalysts: bridging electrochemical and thermal catalysis

**DOI:** 10.1093/nsr/nwag186

**Published:** 2026-04-25

**Authors:** Zhixuan Huang, Yawen Dai, Shuchun Li, Qiwei Duan, Cuiying Jiang, Chongxian Yang, Jianqiang Feng, Mengfei Qiao, Zailai Xie, Jiannian Yao

**Affiliations:** Institute of Molecular Engineering Plus, College of Chemistry, Fuzhou University, Fuzhou 350108, China; Institute of Molecular Engineering Plus, College of Chemistry, Fuzhou University, Fuzhou 350108, China; Key Laboratory of Advanced Carbon-Based Functional Materials (Fujian Province University), Fuzhou University, Fuzhou 350108, China; Key Laboratory of Advanced Carbon-Based Functional Materials (Fujian Province University), Fuzhou University, Fuzhou 350108, China; Institute of Molecular Engineering Plus, College of Chemistry, Fuzhou University, Fuzhou 350108, China; Institute of Molecular Engineering Plus, College of Chemistry, Fuzhou University, Fuzhou 350108, China; Institute of Molecular Engineering Plus, College of Chemistry, Fuzhou University, Fuzhou 350108, China; Institute of Molecular Engineering Plus, College of Chemistry, Fuzhou University, Fuzhou 350108, China; Institute of Molecular Engineering Plus, College of Chemistry, Fuzhou University, Fuzhou 350108, China; Key Laboratory of Advanced Carbon-Based Functional Materials (Fujian Province University), Fuzhou University, Fuzhou 350108, China; Institute of Molecular Engineering Plus, College of Chemistry, Fuzhou University, Fuzhou 350108, China; Key Laboratory of Photochemistry, Institute of Chemistry, Chinese Academy of Sciences, Beijing 100190, China

**Keywords:** nanogalvanic cell catalysts, selective hydrogenation, hydrogenation of nitroaromatics, electrochemical reduction, concerted proton-electron transfer

## Abstract

Selective nitroarene hydrogenation to anilines faces an intractable activity-selectivity trade-off: conventional Horiuti-Polanyi (H-P) catalysis triggers over-hydrogenation of coexisting unsaturated groups via free H* species. Herein, we report Pt@C/TiO_2_ as a nanogalvanic cell catalyst (NGC) that bridges electrochemical and thermal catalysis by integrating concerted proton–electron transfer (CPET) into heterogeneous catalysis. In Pt@C/TiO_2_, Pt nanoparticles wrapped by an ultrathin carbon shell catalyze H_2_ oxidation, whereas TiO_2_ hosts nitro reduction. Protons migrate through solvent hydrogen-bond networks and electrons transfer through the conductive TiO_2_ support, enabling a CPET-like reduction that largely bypasses the H-P route without direct substrate-Pt contact. Electrochemical coupling analyses corroborate galvanic-cell operation. Pt@C/TiO_2_ affords near-quantitative conversion and > 97% selectivity to 4-aminostyrene from 4-nitrostyrene, while the carbon shell blocks CO/sulfur access to Pt, delivering exceptional poisoning tolerance and stability. This NGC paradigm offers a general strategy for site-separated redox catalysis combining electrocatalytic selectivity with thermal-process simplicity.

## Teaser

A nanogalvanic cell catalyst bridges electrochemistry and thermal catalysis by separating hydrogen activation from reduction sites, enabling highly selective production of valuable amines from nitro compounds.

## INTRODUCTION

**Figure 1.**
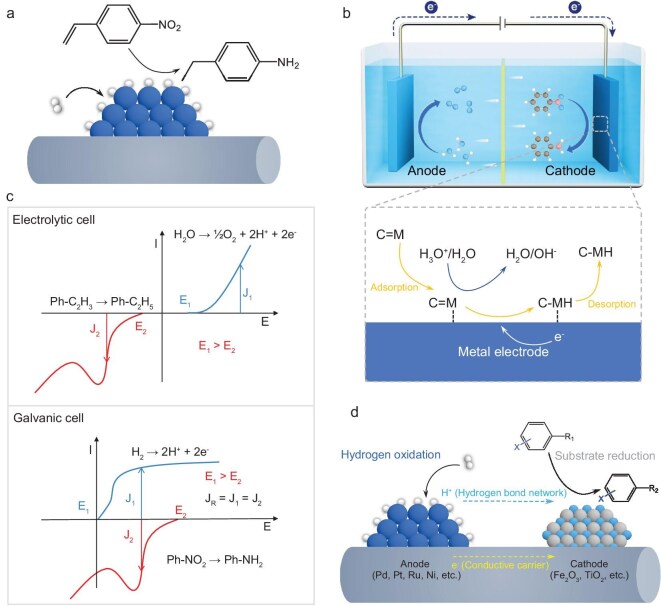
Design of nanogalvanic cell catalysts. (a) Scheme of 4-NS hydrogenation via the H-P mechanism. (b) Scheme of the CPET mechanism in electrocatalytic hydrogenation reactions. (c) Scheme of the relationship between the cathode and anode in an electrolytic cell or galvanic cell. (d) Scheme of the nanogalvanic cell catalyst design.

Catalytic hydrogenation of nitroarenes to anilines underpins the production of high-value aromatic amines and is indispensable to pharmaceuticals, dyes, and fine chemicals [1–5]. However, precise chemoselective reduction of the nitro group (–NO_2_) to the amino group (–NH_2_) in polyfunctionalized nitroarenes remains a long-standing challenge: coexisting reducible functionalities (e.g. C=C, C≡C, carbonyl, cyano) can competitively adsorb and undergo undesired hydrogenation, thereby suppressing formation of the target anilines (Fig. 1a) [6–8]. To fundamentally surmount this long-standing bottleneck, it is imperative to conduct in-depth investigations into the intrinsic reaction mechanism. As articulated in the widely corroborated Horiuti-Polanyi (H-P) mechanism, H_2_ molecules undergo dissociative adsorption at the active sites of metal catalysts, followed by H–H bond cleavage to generate surface-adsorbed hydrogen species (e.g. H* atoms or hydridic H^⁻^) [9–11]. Subsequent adsorption of unsaturated substrates triggers hydrogen abstraction from the catalyst surface, facilitating the hydrogen transfer process and eventual desorption of products—importantly, most pivotal advances in nitroarene hydrogenation documented in the literature to date have been predicated on this mechanistic framework [12,13]. Within this paradigmatic construct, achieving precise selective control hinges on the delicate regulation of substrate adsorption geometry or binding affinity. However, an intrinsic trade-off persists, rendering it extraordinarily challenging to achieve a synergistic balance between exceptional selectivity and superior catalytic activity [14–17]. A classic illustration is the Lindlar catalyst, which enhances selectivity toward anilines by selectively poisoning highly active metal sites (e.g. through modification with PbO or quinoline) to suppress over-hydrogenation and side reactions [18,19]. Regrettably, this sacrificial strategy compromises the intrinsic catalytic activity of the noble metal. It also leads to inefficient utilization of precious metal resources, underscoring the inherent limitations of conventional activity–selectivity modulation paradigms.

In recent years, the rapid advancement of electrocatalytic research has spurred attempts to realize catalytic hydrogenation via electrolysis, with the concerted proton-electron transfer (CPET) mechanism serving as the core for precise chemoselective control [20–22]. Specifically, protons (H^+^, sourced from H_2_O, protic acids or the substrates themselves) and electrons (supplied by the electrode) are synchronously transferred to the substrate; hydrogen species participate in the reaction as a synergistic ‘H^+^ + e^−^’ ensemble, thereby precluding the formation of free atomic hydrogen (H*) intermediates (Fig. 1b) [23,24]. Given the pronounced disparities in reduction potentials among different functional groups, fine-tuning the electrode potential enables facile chemoselective regulation (Fig. 1c)—this unique mechanistic feature underpins ultrahigh selectivity in the hydrogenation of diverse organic substrates (e.g. *α, β*-unsaturated aldehydes to unsaturated alcohols, ketones to secondary alcohols, and nitroarenes to aromatic amines) by eliminating over-hydrogenation induced by free H* [25,26]. More intriguingly, Yang *et al*. discarded the conventional electrolyzer configuration and constructed a galvanic cell system, wherein hydrogen oxidation acts as the anodic reaction and nitroarene reduction as the cathodic counterpart, achieving exceptional chemoselectivity in nitroarene hydrogenation [12]. However, practical electrosynthesis still faces significant obstacles to industrial application, including complex device requirements, fragile ion-exchange membranes that require frequent replacement, and product separation challenges arising from added electrolytes [27,28].

Fundamentally, heterogeneous catalytic reactions are driven by dynamic interactions between reactants, intermediates, products and the catalyst surface. To access target transition states and overcome kinetic barriers, thermal catalysis and electrocatalysis diverge in their core rate-enhancement strategies—the former relies on elevated temperatures, whereas the latter leverages tunable electrode potentials [24]. Conceptually, any heterogeneous surface process involving oxidation state changes, redox transformations, charged species adsorption (even as spectators) or small-molecule heterolytic cleavage shares intrinsic commonalities with electrochemical processes at charged interfaces. The core scientific question is how to implement electrochemical CPET-like chemoselective control within conventional thermal heterogeneous catalysis. In light of these insights, we propose a transformative strategy: integrating the CPET mechanism into traditional heterogeneous catalytic systems. This approach thus harnesses the unparalleled chemoselective control of CPET, while simultaneously circumventing the industrial scalability bottlenecks of electrochemical technologies, offering an elegant solution to the long-standing activity-selectivity trade-off in selective nitroarene hydrogenation (Fig. 1d).

Herein, we present Pt@C/TiO_2_ as the model catalyst—constructed via anchoring platinum nanoparticles (Pt NPs) onto a reducible TiO_2_ support and subsequent conformal carbon shell encapsulation—for the selective hydrogenation of nitrostyrene to aminostyrene. The carbon shell serves as a physical barrier, precluding direct reactant adsorption and hydrogenation on Pt NPs to fully suppress the canonical H-P mechanism. Yet, counterintuitively, the catalyst achieves simultaneous exceptional selectivity and activity—an outcome attributed to the successful establishment of a CPET-driven pathway within the heterogeneous system. Combined systematic characterization and mechanistic investigations delineate the definitive hydrogenation sequence: hydrogen oxidation proceeds preferentially at the carbon-shell-protected Pt active sites; electrons and protons are then synergistically transferred to the TiO_2_ support sites; crucially, nitrostyrene molecules undergo exclusive hydrogenation at TiO_2_ sites without any direct interaction with Pt NPs. We further identify that the electron transport capability of the TiO_2_ support and the hydrogen-bonding network of the reaction solution are pivotal to facilitating this synergistic proton-electron transfer. Notably, this unique mechanistic paradigm endows the catalyst with robust applicability across the selective hydrogenation of diverse aromatic amine precursors, coupled with remarkable resistance to sulfur and CO poisoning [29,30]. Analogous to the anodic and cathodic reactions in a conventional galvanic cell, this spatially segregated dual-site system—with H_2_ oxidation and nitroarene hydrogenation confined to distinct active domains—is formally designated a nanogalvanic cell catalyst (NGC). Unlike device-based ‘noncontact’ hydrogenation concepts implemented in H-cell configurations, which separate H_2_ and substrate in different compartments and require electrochemical hardware, our approach integrates the paired redox half-reactions within a single heterogeneous catalyst architecture.

## RESULTS AND DISCUSSION

### Structural design and characterization of Pt@C/TiO_2_ catalysts

**Figure 2.**
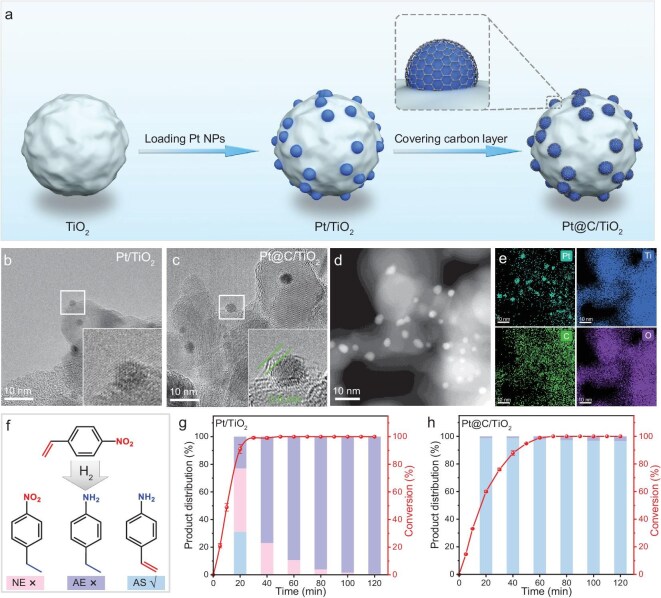
Catalyst with spatially separated sites enables selective hydrogenation. (a) Schematic diagram of catalyst synthesis. (b and c) AC-TEM images for the Pt/TiO_2_ (b) and Pt@C/TiO_2_ (c). (d) STEM-HAADF image, and (e) EDS elemental mapping images for the Pt@C/TiO_2_. (f) Scheme of hydrogenation of 4-NS. (g and h) Time-dependent catalysis by Pt/TiO_2_ (g) and Pt@C/TiO_2_ (h). Reaction conditions: 60°C, 1 bar H_2_, 0.001 mol of Pt/mol of each substrate, 10 mL ethanol as solvent.

The Pt@C/TiO_2_ catalyst was synthesized by depositing Pt onto TiO_2_ via impregnation reduction, followed by carbon shell formation through vapor-phase deposition (Fig. 2a). H_2_PtCl_6_ and NaBH_4_ were used as the metal precursor and reducing agent, respectively, to reduce and uniformly deposit Pt NPs onto anatase TiO_2_, yielding the Pt/TiO_2_ precursor. Transmission electron microscopy (TEM) images confirmed the uniform distribution of Pt NPs without noticeable agglomeration (Fig. 2b and Fig. S1). The final Pt@C/TiO_2_ catalyst was obtained by depositing a carbon shell onto the Pt surface via vapor-phase deposition at 400 °C under a nitrogen atmosphere. Aberration-corrected transmission electron microscopy (AC-TEM) images showed that the Pt NPs were encapsulated by a carbon shell approximately 0.6 nm thick (Fig. 2c). High-angle annular dark-field imaging (HAADF) and energy dispersive spectroscopy (EDS) elemental mapping further confirmed the uniform dispersion of Pt NPs on the TiO_2_ surface and their encapsulation by the carbon shell (Fig. 2d, e). X-ray diffraction (XRD) patterns indicate that no new phases were formed after vapor deposition, indicating that TiO_2_ did not undergo phase transformation and that Pt particles did not undergo severe aggregation (Fig. S2). X-ray photoelectron spectroscopy (XPS) revealed Pt 4f peaks at 73.7 eV and 70.3 eV (Fig. S3d), and peak deconvolution indicated that Pt existed predominantly in the metallic state [31–33]. The C 1 s and Ti 2p spectra also confirmed the successful formation of the carbon shell (Fig. S4a, d).

### Enhanced chemoselectivity in 4-nitrostyrene hydrogenation

To probe catalytic selectivity in hydrogenation reactions, 4-nitrostyrene (4-NS), featuring both a nitro group (–NO_2_) and a conjugated C=C group, was selected as a model substrate (Fig. 2f). When subjected to Pt/TiO_2_ catalysis, the high intrinsic reactivity but poor selectivity of Pt toward both functionalities led to the formation of multiple products, including 4-aminostyrene (4-AS), 4-nitroethylbenzene (4-NE), and 4-ethylaniline (4-AE) (Fig. 2g). Although 4-NS was completely converted within 30 minutes, the selectivity toward the desired product 4-AS was low at 11.27% and decreased further with prolonged reaction time, indicating significant over-hydrogenation. In stark contrast, the Pt@C/TiO_2_ catalyst exhibited markedly enhanced chemoselectivity under identical conditions (Fig. 2h). Even after prolonging the reaction time to 70 minutes, 4-NS remained fully converted, and the selectivity toward the main product 4-AS reached as high as 97.36%. Notably, both catalysts showed comparable activity for nitrobenzene hydrogenation, whereas Pt@C/TiO_2_ significantly inhibited the hydrogenation of styrene (Fig. S5).

### Role of carbon shell in site-selective catalysis

**Figure 3.**
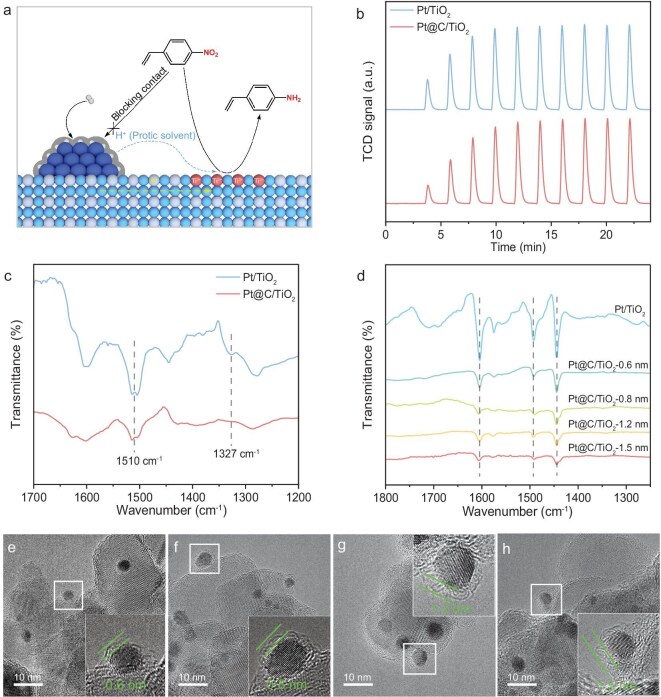
The role of carbon shells in catalytic processes. (a) Scheme diagram of 4-NS hydrogenation over Pt@C/TiO_2_. (b) The TCD signal of H_2_ impulse over different catalysts. (c) FTIR spectra of adsorbed 4-NS at the Pt/TiO_2_ and Pt@C/TiO_2_ after heat treatment at 200°C. (d) FTIR spectra of adsorbed pyridine after desorption at Pt/TiO_2_, Pt@C/TiO_2_-0.6 nm, Pt@C/TiO_2_-0.8 nm, Pt@C/TiO_2_-1.2 nm and Pt@C/TiO_2_-1.5 nm after heat treatment at 200°C. (e–h) AC-TEM images of the Pt@C/TiO_2_-0.6 nm (e), Pt@C/TiO_2_-0.8 nm (f), Pt@C/TiO_2_-1.2 nm (g) and Pt@C/TiO_2_-1.5 nm (h).

Given that the observed selective differences are directly related to the presence of the carbon shell, we first focused on studying their role in the catalytic process by evaluating the catalytic activity of Pt/TiO_2_ and Pt@C/TiO_2_ on nitrobenzene and styrene. Under a hydrogen atmosphere, styrene readily undergoes hydrogenation solely upon contact with Pt NPs or Pd NPs [34,35]. However, the Pt@C/TiO_2_ catalyst cannot catalyze styrene hydrogenation (Fig. S5b), indicating that the carbon shell effectively blocks direct contact between the substrate and the Pt active sites [36]. However, H_2_ pulse adsorption experiments confirm that the carbon shell does not significantly hinder hydrogen adsorption, indicating that Pt@C/TiO_2_ retains its ability to activate hydrogen (Fig. 3b). This indicates that, despite this barrier, the smaller size of H_2_ molecules allows them to penetrate the carbon shell and reach the Pt nanoparticles [37]. Based on these experimental results, we hypothesize that a unique reduction mechanism for nitro groups may exist: hydrogen first penetrates the carbon shell, diffuses to the Pt nanoparticle surface for activation, and generates adsorbed hydrogen atoms. These adsorbed hydrogen atoms then dissociate into protons and electrons, with electrons being transferred to the TiO_2_ sites via conductive carriers, while protons migrate through the protic solvent, thereby enabling the reduction of nitro groups (Fig. 3a). Fourier transform infrared (FTIR) spectroscopy was employed to investigate the effect of the carbon shell on the adsorption of 4-NS. The results showed that both Pt/TiO_2_ and Pt@C/TiO_2_ could adsorb 4-NS (Fig. 3c). Pyridine adsorption FTIR spectra (Fig. 3d) revealed the presence of numerous exposed acidic sites on the Pt@C/TiO_2_ surface, suggesting that TiO_2_ serves as the key active site for 4-NS adsorption and selective hydrogenation [38].

To further investigate the effect of the carbon shell on substrate adsorption and catalytic performance, a series of Pt@C/TiO_2_ catalysts with tunable carbon shell thicknesses were prepared by using varying volumes of ethanol (0.5, 1, 3, and 5 mL) as the carbon source. AC-TEM images showed that the carbon shell thickness increased with increasing ethanol dosage. The measured thicknesses of the carbon shells were 0.6, 0.8, 1.2 and 1.5 nm, respectively (Fig. 3e–h). Therefore, we named the resulting catalysts as Pt@C/TiO_2_-0.6 nm, Pt@C/TiO_2_-0.8 nm, Pt@C/TiO_2_-1.2 nm and Pt@C/TiO_2_-1.5 nm. Correspondingly, the catalytic activity for –NO_2_ hydrogenation exhibited a clear downward trend (Fig. S6). The decrease in catalytic performance can be attributed to the thickened carbon shells, which hinder hydrogen diffusion and consequently reduce hydrogen activation. Hydrogen temperature-programmed desorption (H_2_–TPD) experiments indicate that the amount of hydrogen desorption decreases with increasing carbon shell thickness (Fig. S7). Additionally, the carbon shell also partially covers the TiO_2_ surface at high deposition dosages, inhibiting the effective adsorption of 4-NS onto the TiO_2_ surface, thus suppressing the hydrogenation reaction. This phenomenon is further supported by pyridine adsorption FTIR analysis (Fig. 3d) [39].

### Proton-electron transfer mechanism

**Figure 4.**
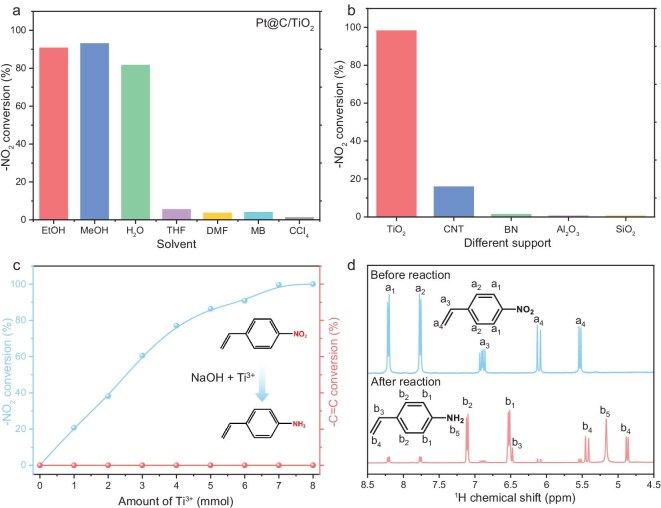
Importance of both proton and electron transfer channels. (a) Conversion of 4-NS over Pt@C/TiO_2_ at 30 min under different solvents. Reaction conditions: 60°C, 1 bar H_2_, 0.001 mol of Pt/mol of each substrate,10 mL solvent. (b) Conversion of 4-NS over Pt@C catalysts with different supports at 30 min. Reaction conditions: 60°C, 1 bar H_2_, 0.001 mol of Pt/mol of each substrate,10 mL ethanol as solvent. (c) Conversion of –NO_2_ and C=C group as a function of Ti^3+^ consumption. (d) ^1^H-NMR spectra of residual proton signals of reference incompletely deuterated DMSO-d6 before and after the reduction of 4-nitrostyrene by TiCl_3_.

Since 4-NS molecules cannot penetrate the carbon shell to directly contact the Pt NPs, hydrogen activation and substrate reduction in the Pt@C/TiO_2_ system become spatially decoupled. The process is evidenced as H_2_ is activated at the Pt sites, while the reduction of 4-NS occurs on the TiO_2_ surface. This site-separated catalytic pathway is also supported by the difference in apparent activation energy between Pt/TiO_2_ and Pt@C/TiO_2_ (Fig. S8) [40]. To further explore the migration mechanism of hydrogen-active species, the catalytic hydrogenation of 4-NS over Pt@C/TiO_2_ was examined in solvents of different polarity. Here, the solvent set spans protic, highly polar media (H_2_O, MeOH and EtOH) that can donate protons and form extended hydrogen-bond networks, polar aprotic solvents (DMF and THF) with limited/absent proton-donating ability, and low-polarity/nonprotic solvents (methyl benzoate and CCl_4_). The catalyst exhibited high activity in protic solvents but was nearly inactive in aprotic solvents (Fig. 4a), indicating that active hydrogen species were transferred in the form of protons via solvent mediation [41,42]. Additionally, in EtOH/THF mixed solvents with varying volume ratios, catalytic activity increased with higher EtOH content (Fig. S9), further confirming the promoting effect of the proton donor on the reaction. When deuterated methanol (CD_3_OD) was used as the solvent, Pt@C/TiO_2_ exhibited a more pronounced isotope effect than Pt/TiO_2_ (Fig. S10), indicating that its reaction pathway is highly sensitive to proton transfer. In summary, these results demonstrate that active hydrogen species migrate from Pt to TiO_2_ sites in the form of protons, thereby enabling the selective hydrogenation of 4-NS.

In addition to proton transfer, efficient electron transfer from Pt NPs to TiO_2_ is also critical for achieving selective hydrogenation of 4-NS. To verify the role of TiO_2_ in electron transfer, four Pt@C catalysts with different supports were synthesized using the same vapor-phase deposition method: Pt@C/SiO_2_ (Fig. S11), Pt@C/BN (Fig. S12), Pt@C/Al_2_O_3_ (Fig. S13), and Pt@C/CNT (Fig. S14). Owing to the excellent electronic conductivity of TiO_2_, Pt@C/TiO_2_ provides an efficient electron transfer pathway, resulting in significantly higher catalytic activity than the other non-reducible support catalysts (Fig. S15 and Fig. 4b). Furthermore, physical mixing of Pt@C/Al_2_O_3_ with TiO_2_ did not significantly improve catalytic activity (Fig. S16), suggesting that effective electron transfer requires direct contact and structural continuity between Pt and TiO_2_.

Notably, although CNTs possess electronic conductivity and can facilitate electron transfer, the catalytic activity of Pt@C/CNT remains inferior to that of Pt@C/TiO_2_(Fig. 4b). This observation indicates that TiO_2_ not only serves as an electron conduit but also directly participates in the redox process. When electrons migrate from Pt NPs to the TiO_2_ surface, a portion of Ti^4+^ is reduced to Ti^3+^, accompanied by the formation of oxygen vacancies that selectively adsorb –NO_2_ groups (Fig. S17) [43]. Subsequent electron and proton transfer steps then drive the reduction of nitro groups to amino groups. To further confirm the role of Ti^3+^ in nitro group reduction, varying amounts of TiCl_3_ were added to a mixed 4-NS/NaOH reaction system (Fig. S18). The results showed that as the amount of Ti^3+^ increased, the conversion of –NO_2_ groups steadily improved, while the C=C group remained intact throughout the reaction (Fig. 4c), further confirming the selective reduction capability of Ti^3+^ toward nitro groups. Beyond Ti^3+^, oxygen vacancies on TiO_2−x_ (often correlated with Ti^3+^-rich surfaces) can serve as electron-rich defect sites that strengthen nitro-group adsorption/polarization, thereby facilitating selective –NO_2_ reduction [44]. In addition, the promotion effect may not be exclusive to Ti³⁺, but could more broadly arise from reduced, redox-active oxide centers that provide electron-rich sites for nitro-group activation, as further supported by the analogous reactivity observed for Fe^2+^ and Ce^3+^ containing systems (Fig S19).

### Galvanic cell-like catalytic pathway

Based on the above mechanistic insights, the hydrogenation of nitro compounds over Pt@C/TiO_2_ can be viewed as a galvanic cell-like process composed of two spatially separated half-reactions. In this system, Pt NPs serve as the anode for the hydrogen oxidation reaction (HOR), while TiO_2_ functions as the cathode for the nitro group reduction reaction (–NO_2_RR). To validate this mechanism, linear sweep voltammetry (LSV) measurements were conducted for each half-reaction using a three-electrode system (Fig. S20). Pt-loaded carbon paper was used as the working electrode for HOR (Fig. S21), while TiO_2_-coated carbon paper was used for –NO_2_RR (Fig. S22). All measurements were conducted under H_2_ or N_2_ atmospheres to eliminate interference from air (Fig. S20a). In the absence of nitro compounds, the TiO_2_/carbon paper electrode exhibited a reduction current onset at 0.47 V relative to the blank carbon paper electrode (Fig. S20b), attributable to the reduction of Ti^4+^ to Ti^3+^. Upon addition of 4-NS, a more pronounced reduction current was observed on the TiO_2_/carbon paper electrode, with an onset potential of 0.64 V. This enhanced current was attributed to the reduction of the –NO_2_ group rather than the C=C group, as the addition of styrene did not induce a significant current response (Fig. S20c). Moreover, the onset potential for –NO_2_RR on the TiO_2_ electrode was more positive than that for HOR on the Pt electrode (Fig. S20d), suggesting that a spontaneous galvanic process is thermodynamically feasible. Accordingly, the intersection of the HOR and –NO₂RR polarization curves can be regarded as the putative short-circuit operating point, where anodic and cathodic currents are balanced (Fig. S23).

**Figure 5.**
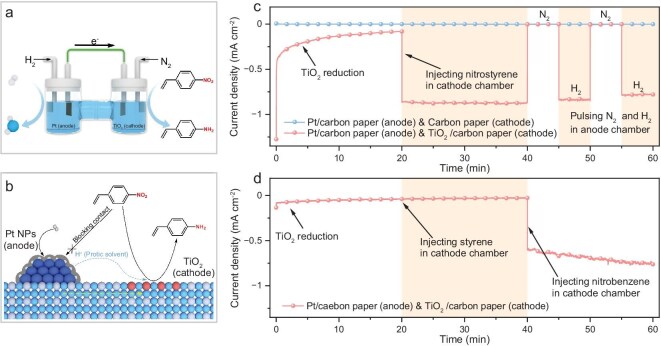
Structural model and electrochemical verification of the nanogalvanic cell catalyst. (a and b) Scheme of the galvanic cell model and catalyst structure. (c and d) Short-circuit current generated as a function of time using a two-electrode system. Reaction conditions: 0.1 M KOH as electrolyte, Pt/carbon-paper electrode as anode under N_2_ or H_2_, TiO_2_/carbon-paper electrode or clean carbon-paper electrode as cathode under N_2_.

Based on the above findings, a two-chamber electrochemical cell was constructed by coupling the two half-reactions. The Pt/carbon paper electrode served as the anode for HOR, while the TiO_2_/carbon paper electrode acted as the cathode for –NO_2_RR. Hydrogen gas was injected into the anode reaction chamber as the reaction gas, and nitrogen gas was injected into the cathode reaction chamber as the protective gas (Fig. 5a). In the absence of substrate, a gradually decreasing current was observed during the initial 10 minutes, originating from HOR and the reduction of surface Ti^4+^ on TiO_2_ (Fig. 5c). Once the current stabilized (after ∼20 minutes), 1 mmol of 4-NS was added to the cathodic chamber, leading to a sharp increase in short-circuit current due to the electrochemical potential difference between the two electrodes. At 40 minutes, intermittently replacing H_2_ with N_2_ in the anode chamber caused the short-circuit current to vanish, confirming that HOR directly contributed to current generation. To verify whether the short-circuit current originated from –NO_2_ reduction rather than C=C hydrogenation, nitrobenzene and styrene were used as control substrates. A significant current was observed only in the presence of nitrobenzene, whereas styrene failed to induce any noticeable current response (Fig. 5d).

Based on these results, the galvanic cell model (Fig. 5a) and the catalyst architecture (Fig. 5b) establish a conceptual bridge between electrocatalytic principles and thermal catalytic processes. The electrochemical cell validates the thermodynamic viability of spatially segregated half-reactions, hydrogen oxidation at the Pt anode and nitro group reduction at the TiO_2_ cathode. This configuration mirrors the Pt@C/TiO_2_ catalyst design, where the carbon shell acts as a physical separator, analogous to an electrolyte barrier in a galvanic cell, ensuring that hydrogen activation (anode-like site) and substrate reduction (cathode-like site) occur at spatially distinct locations. Consequently, the hydrogenation of –NO_2_ on Pt@C/TiO_2_ follows an unconventional pathway, where the nitro group is reduced without direct contact with the Pt surface, avoiding competitive hydrogenation of the C=C bond. This spatial decoupling mechanism, driven by CPET, provides precise control over the reaction pathway and suppresses the hydrogenation of other unsaturated functional groups. By mimicking a nanogalvanic cell, this design enables selective hydrogenation across a broad substrate scope (Figs S24–S30 and Table S1) and demonstrates the inherent electrochemical mechanism of the thermal hydrogenation process.

### Resistance to CO and sulfur poisoning

**Figure 6.**
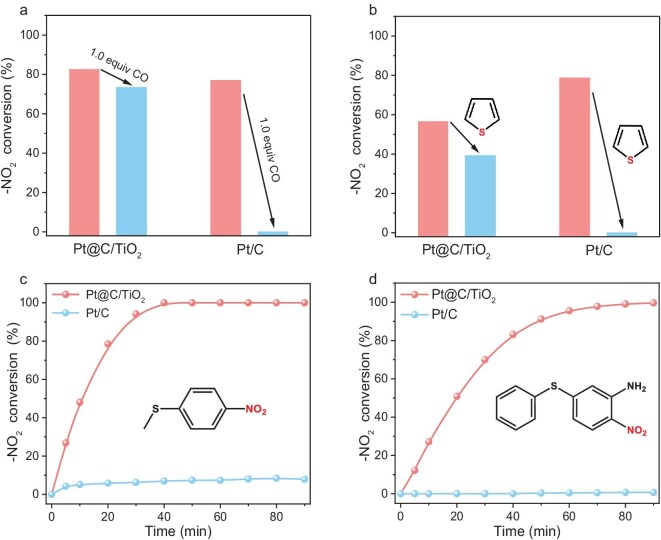
Anti CO and sulfur toxicity experiments in –NO_2_ hydrogenation. (a) The conversion comparison of Pt@C/TiO_2_ and Pt/C before and after CO poisoning. (b) The conversion comparison of Pt@C/TiO_2_ and Pt/C before and after thiophene poisoning. (c) Time-dependent catalysis of the 4-nitrothioanisole by the Pt@C/TiO_2_ and Pt/C. (d) Time-dependent catalysis of 2-nitro-5-(phenylthio)aniline by the Pt@C/TiO_2_ and Pt/C. Reaction conditions: 60°C, 1 bar (b) or 3 bar (c and d) H_2_, 1 mmol of substrate, 19.5 mg (b) or 39 mg (c and d) of samples and 10 mL ethanol as solvent.

CO poisoning is a common challenge in catalytic hydrogenation, particularly with noble metal catalysts, as CO binds strongly to the catalyst surface and is difficult to desorb, leading to catalyst deactivation [45]. Owing to the protective carbon shell, the Pt@C/TiO_2_ catalyst exhibits excellent CO tolerance. The carbon shell effectively prevents direct contact between Pt NPs and CO molecules, thereby mitigating catalyst poisoning. This was supported by CO adsorption FTIR spectra, which showed strong CO adsorption on Pt/TiO_2_ but minimal adsorption on Pt@C/TiO_2_ (Fig. S31). Comparative hydrogenation experiments were conducted by introducing 1.0 equivalent of CO into the 4-NS hydrogenation system. While commercial Pt/C catalyst was completely deactivated, Pt@C/TiO_2_ retained substantial catalytic activity (Fig. 6a). Furthermore, the Pt@C/TiO_2_ catalyst exhibited excellent recyclability, maintaining high activity over multiple catalytic cycles (Fig. S32).

In addition to its resistance to CO poisoning, Pt@C/TiO_2_ also demonstrates significant resistance to sulfur-containing substrates, which are typically known to poison traditional platinum catalysts [46]. To investigate this, we introduced thiophene, a compound that is known to poison traditional precious metal catalysts, into the hydrogenation system of 4-nitrostyrene [47,48]. The results revealed that, compared to the commercial Pt/C catalyst, Pt@C/TiO_2_ exhibited remarkable anti-poisoning properties (Fig. 6b). Furthermore, we evaluated the catalytic performance of Pt/C and Pt@C/TiO_2_ for the hydrogenation of 4-nitrobenzyl methyl sulfide and 2-nitro-5-(phenylthio)aniline (Fig. 6c, d). Notably, 2-nitro-5-(phenylthio)aniline is a key intermediate in the synthesis of the anthelmintic drug fenbendazole. As expected, the commercial Pt/C catalyst was almost completely deactivated, whereas Pt@C/TiO_2_ effectively resisted the poisoning effect of thiols (Figs S33 and S34), highlighting its potential for applications in the pharmaceutical industry.

## CONCLUSION

In conclusion, we report a nanogalvanic cell catalyst Pt@C/TiO_2_ that enables a spatially decoupled hydrogenation mechanism, in which H_2_ activation and substrate reduction occur at distinct sites. The carbon shell physically prevents substrate molecules from directly accessing the Pt surface while allowing hydrogen permeation, thereby isolating the HOR and –NO_2_RR half-reactions. TiO_2_, beyond serving as an electron acceptor, acts as the catalytic site for –NO_2_ reduction, facilitated by its high conductivity, redox activity, and ability to stabilize intermediate species. Mechanistic investigations confirm that the reaction follows a proton–electron transfer pathway analogous to a galvanic cell. This conceptually distinct design affords excellent chemoselectivity toward nitro groups while leaving other reducible groups untouched, and offers a generalizable strategy for constructing site-separated catalytic systems with spatially decoupled redox processes. In addition, the physical isolation of Pt by the carbon shell endows the catalyst with excellent tolerance to poisoning species such as CO and thiols, enabling selective hydrogenation of functionalized nitroarenes under demanding conditions. We envision that this galvanic-mode catalytic paradigm may serve as a blueprint for designing advanced heterogeneous systems with programmable redox pathways.

## Supplementary Material

nwag186_Supplemental_File
