# Supplementary material for: Nanogalvanic cell catalysts: bridging electrochemical and thermal catalysis
Source: Natl Sci Rev. 2026 Apr 25;13(12):nwag186. doi: 10.1093/nsr/nwag186 (PMC13317451; doi:10.1093/nsr/nwag186)
Supplement: nwag186_Supplemental_File [file nwag186_supplemental_file.pdf]

Supplemental Information

Nanogalvanic Cell Catalysts: Bridging Electrochemical and Thermal Catalysis

Materials and methods

Chemicals and materials. Chloroplatinic acid hexahydrate ($H_2PtCl_6·6H_2O$, Pt 37.5%), potassium hydroxide (KOH 90%) potassium bromide (KBr SP), boron nitride (BN <150nm 99.8%), tetrahydrofuran (THF Water≤30 ppm), nitrobenzene ($C_6H_5NO_2$ 99%), styrene (C_8H_8 99.5%), 4-nitrostyrene ($C_8H_7NO_2$ 98%), Methanol-d4 (D, 99.8%) and dimethyl sulfoxide-d6 (D, 99.8%), was purchased from Shanghai Adamas Reagent Co. Ltd. Methanol (CH_3OH, AR), ethanol (C_2H_5OH, AR), N, N-dimethylformamide (DMF, AR), methylbenzene (C_7H_8, AR), carbon tetrachloride (CCl_4, AR) and sodium borohydride ($NaBH_4$, AR) were purchased from Sinopharm Chemical Reagent Co. Ltd (China). Aluminum oxide (Al_2O_3 30nm 99.99%) and fumed silica (SiO_2 50nm 99.8%), were purchased from Shanghai McLean Biochemical Technology Co. Ltd. Carbon nanotube (CNTs) and titanium trichloride ($TiCl_3$ 15-20%) were purchased from Shanghai Aladdin Biochemical Technology Co. Ltd. Titanium tetrachloride ($TiCl_3$ 99.9%) were purchased from Anhui Zesheng Technology Co. Ltd. P25 titanium dioxide (TiO_2 P25) were purchased from Degussa Chemicals (Shanghai) Co. Ltd. H_2 (99.999%), Ar (99.999%), 5% CO/Ar and N_2 (99.999%) were purchased from Fuzhou General Gas Co. Ltd. All the water used was DI water.

Methods

Preparation of Pt@C/TiO_2-1mL, Pt@C/TiO_2-3mL and Pt@C/TiO_2-5mL catalysts. Pt@C/TiO_2-1mL, Pt@C/TiO_2-3mL and Pt@C/TiO_2-5mL catalysts were prepared using a similar method as for the Pt@C/TiO_2 catalysts, but with 1 ml, 3 ml and 5 ml of ethanol as the carbon source, respectively.

Preparation of Pt@C/BN, Pt@C/Al_2O_3, Pt@C/SiO_2 and Pt@C/CNT. Pt@C/BN, Pt@C/Al_2O_3, Pt@C/SiO_2 and Pt@C/CNT catalysts were prepared using a similar method to that used for Pt@C/TiO_2 catalysts, but with BN, Al_2O_3, SiO_2 and CNT as the carriers of Pt nanoparticles.

Characterization

Transmission electron microscopy (TEM). TEM and energy dispersive spectroscopy (EDS) elemental mapping measurements were performed on a Talos F200s transmission electron microscope operating at 200 kV. The samples were prepared by dropping ethanol dispersion of samples onto 300-mesh carbon-coated copper grids and immediately evaporating the solvent.

Aberration-corrected transmission electron microscopy (AC-TEM). AC-TEM and energy dispersive spectroscopy (EDS) elemental mapping measurements were performed on a JEOL JEM-ARM300 F2 aberration-corrected transmission electron microscope operating at 300 kV. The samples were prepared by dropping ethanol dispersion of samples onto 300-mesh carbon-coated copper grids and immediately evaporating the solvent.

Powder X-ray diffraction (XRD). The XRD patterns were measured by Rigaku Ultima IV using Cu Kα radiation. The operation voltage and current were 40 kV and 30 mA, respectively. The scanning speed was set as 10 degree·min^{-1}.

X-ray photoelectron spectroscopy (XPS). XPS spectra were recorded by a ESCALAB QXI X-ray photoelectron spectroscope using a monochromatic Al Kα line source (hv = 1486.69 eV), which was operated at 15 kV and 300 W. The pass energy was 35 eV, and the base pressure of the analysis chamber was 5.0×10^{-9} mbar. The signal of C 1s peak at 284.8 eV was used for energy calibration.

Gas chromatography-mass spectrometry (GC-MS) measurements. GC-MS were recorded on a gas chromatography mass spectrometry (GCMS-QP2010 SEW).

Nuclear Magnetic Resonance (NMR) measurements. 1H NMR spectra were acquired using a Bruker Ascend 600 MHz NMR spectrometer equipped with a cryoprobe CPTCI. Chemical shifts were referenced to the residual proton signal of incompletely deuterated DMSO-d6. 200 μL of the sample solution were mixed with 500 μL of DMSO-d6 in an NMR tube for NMR testing.

Hydrogen chemisorption measurements. Hydrogen chemisorption experiments (pulse mode) were performed at 333 K on a BelCata II chemical adsorption instrument with TCD detector. 100 mg catalysts were used in the measurements. Before experiments, the catalysts was treated at 473 K for 1 h and then cooled to 333 K under the Ar flow (30 mL min^{-1}). Then, the exact 1 mL pulse of 5% H_2/Ar was delivered to the reactor (30 mL min^{-1}), and the time between pulses was 2 min.

Diffuse reflectance infrared Fourier transform spectroscopy (DRIFTS). The in-situ Fourier transform infrared spectroscopy (FTIR) was carried out on a Thermo Fisher IS50 spectrometer equipped with a Harrick Scientific DRIFT cell and a mercury cadmium telluride (MCT) detector. DRIFT spectra were collected in the range of 4000 - 650 cm^{-1}, accumulating 32 scans at 4 cm^{-1} resolution. For the in-situ 4-nitrostyrene (4-NS) adsorption on Pt/TiO_2 and Pt@C/TiO_2, the samples were loaded into the in-situ

chamber and treated with 5% H_2/Ar gas (30 mL min^{-1}) for 20 minutes at 60 °C. The gas flow was then switched to Ar gas (30 mL min^{-1}), warmed to 200 °C, and purged for 60 minutes. The temperature was then reduced to 30°C and background spectra were collected for background correction. Then, 4-nitrostyrene (4-NS) was brought into the chamber with Ar gas (30 mL min-1) for 60 minutes before being introduced into the chamber. The temperature was then switched to Ar gas (30 mL min^{-1}) and increased to 200°C, purged for 60 min, cooled to 30°C, and the in-situ FTIR spectra were recorded. For the in-situ pyridine adsorption on Pt/TiO_2 and Pt@C/TiO_2, the test method adopted was consistent with the adsorption of 4-NS except that 4-NS was replaced by pyridine. For the in-situ CO adsorption on Pt/TiO_2 and Pt@C/TiO_2, the samples were loaded into the in-situ chamber and treated with 5% H_2/Ar gas (30 mL min^{-1}) for 20 minutes at 60 °C. The gas flow was then switched to Ar gas (30 mL min^{-1}), warmed to 200 °C, and purged for 60 minutes. The temperature was then reduced to 30°C and background spectra were collected for background correction. Then, switch to 5% CO/Ar gas was introduced into the chamber for 60 minutes to ensure that the adsorption was saturated. The temperature was then switched to Ar gas (30 mL min^{-1}) and increased to 200°C, purged for 60 min, cooled to 30°C, and the in-situ FTIR spectra were recorded.

Electrochemical characterization

Electrode fabrication. To prepare the Pt/carbon paper electrode (1mg cm^{-2} for Pt loading), 100 μL of chloroplatinic acid solution (0.051 M) was dropped onto a 1 × 1 cm piece of pretreated carbon paper. After drying, 500 μL of 0.1 M $NaBH_4$ was slowly added dropwise for reduction. To prepare the TiO_2/carbon paper electrode (1mg cm^{-2} for TiO_2 loading), 100 μL of hydrochloric acid solution of titanium tetrachloride (0.053 M) was dropped onto a 1 × 1 cm piece of pretreated carbon paper. Then 500 μL of 0.1 M NaOH was slowly added dropwise.

Electrochemical measurements. All electrochemical measurements were performed on Ivium-n-Stat electrochemical workstation in a H-cell (Wuhan Gaossunion Technology Co. Ltd.) in 0.1 M KOH aqueous solution at 25 °C.

linear scan voltammetry (LSV) curves for both of the nitrogroup reduction reaction (NO_2RR) and hydrogen oxidation reaction (HOR) were tested in a three-electrode system under N_2 gas (30 mL min^{-1}) protection and recorded with a scan rate of 10 mV s^{-1}. The Pt/carbon paper electrode or the TiO_2/Carbon-paper electrode was used as the working electrode, and a Hg/HgO/OH electrode and a carbon rod as the reference

electrode and the counter electrode, respectively. The presented potential values were calibrated to a reversible hydrogen electrode (RHE) and the equation is: E(RHE) = E(Hg/HgO) + 0.097 + pH × 0.0592.

The primary cell experiments were performed in the H-cell using a two-electrode system, in which the -NO_2RR and HOR occurs at the cathode and anode respectively. The TiO_2/carbon paper electrode and Pt/carbon paper electrode were placed in cathode and anode chamber respectively. The cathode and anode chambers are separated by an anion exchange membrane, and protected by H_2 and N_2 gas (30 mL min^{-1}) respectively. The i-t curves were recorded by short circuit of the cathode and anode. A certain amount of reactants (1mmol) were added into the cathode chamber under magnetic stirring (900 rpm) after the current stabilized for 20 min to record the primary cell reaction process. After addition of the reactants for 20 min, a pulsed gas of H_2 or N_2 (30 mL min^{-1}) was supplied in the anode chamber to record the current changes.

Supplementary Figures and Tables

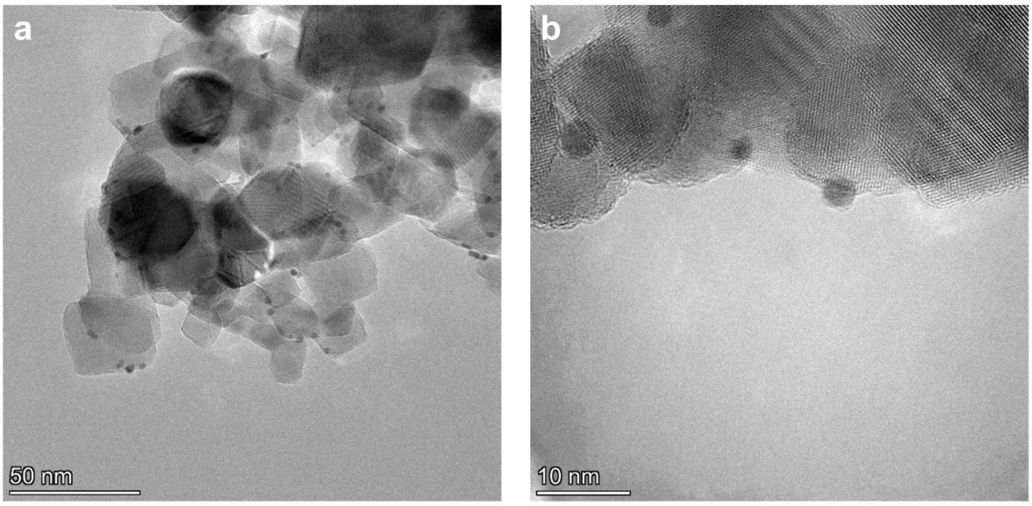

Figure S1. TEM images of the Pt/TiO_2.

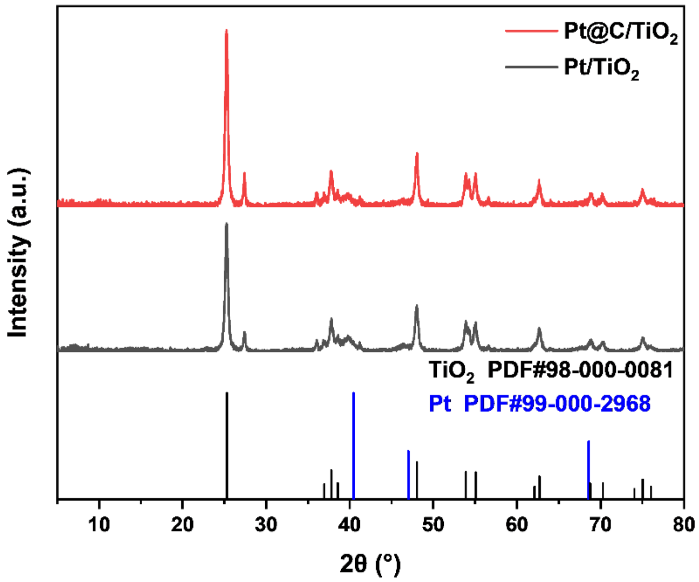

Figure S2. XRD pattern of Pt/TiO_2 and Pt@C/TiO_2.

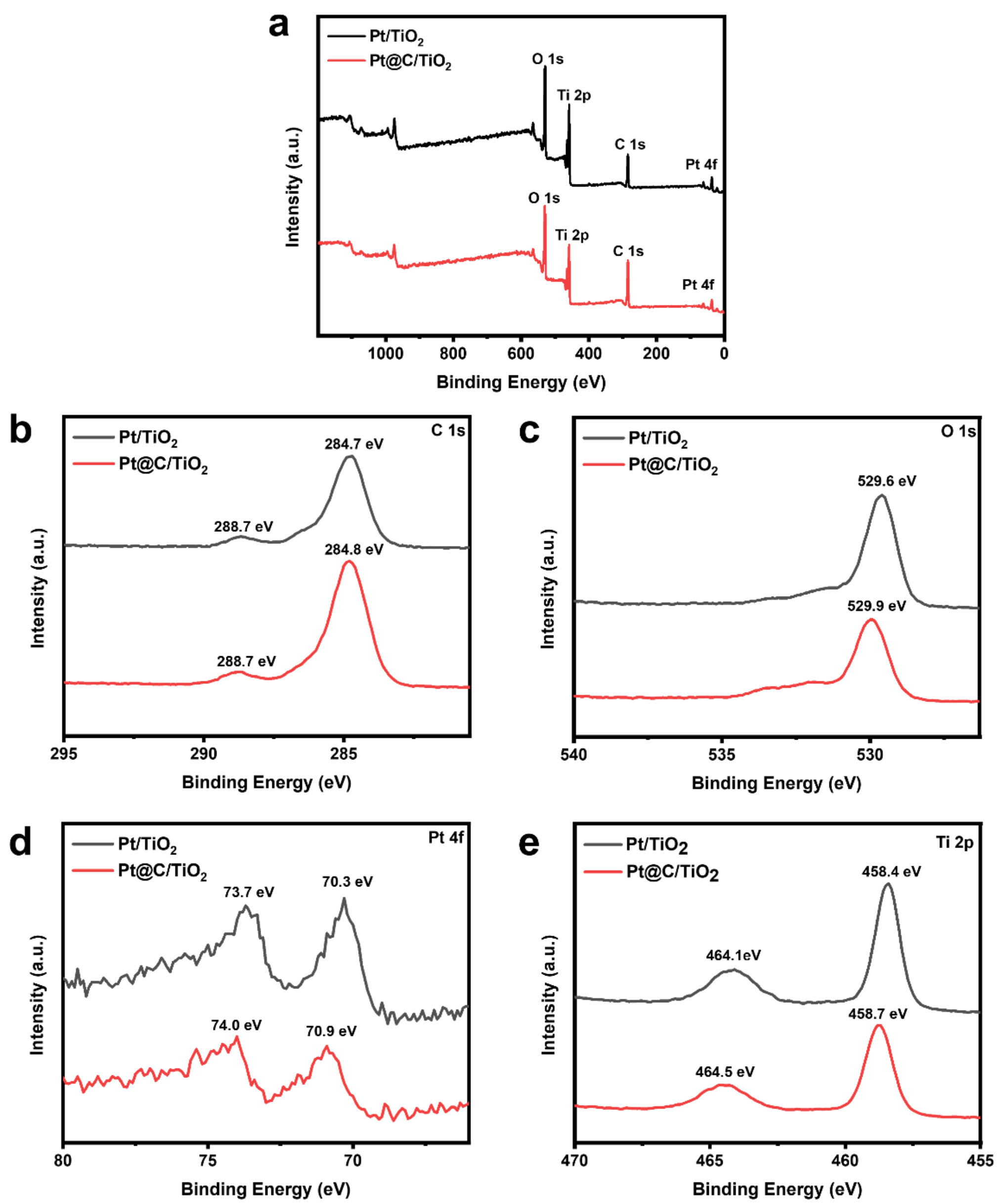

Figure S3. XPS analytic result of Pt/TiO_2 and Pt@C/TiO_2. (a) The wide-range XPS spectra of samples; (b) XPS core-level spectra of C 1s; (c) XPS core-level spectra of O 1s; (d) XPS core-level spectra of Pt 4f; (E) XPS core-level spectra of Ti 2p.

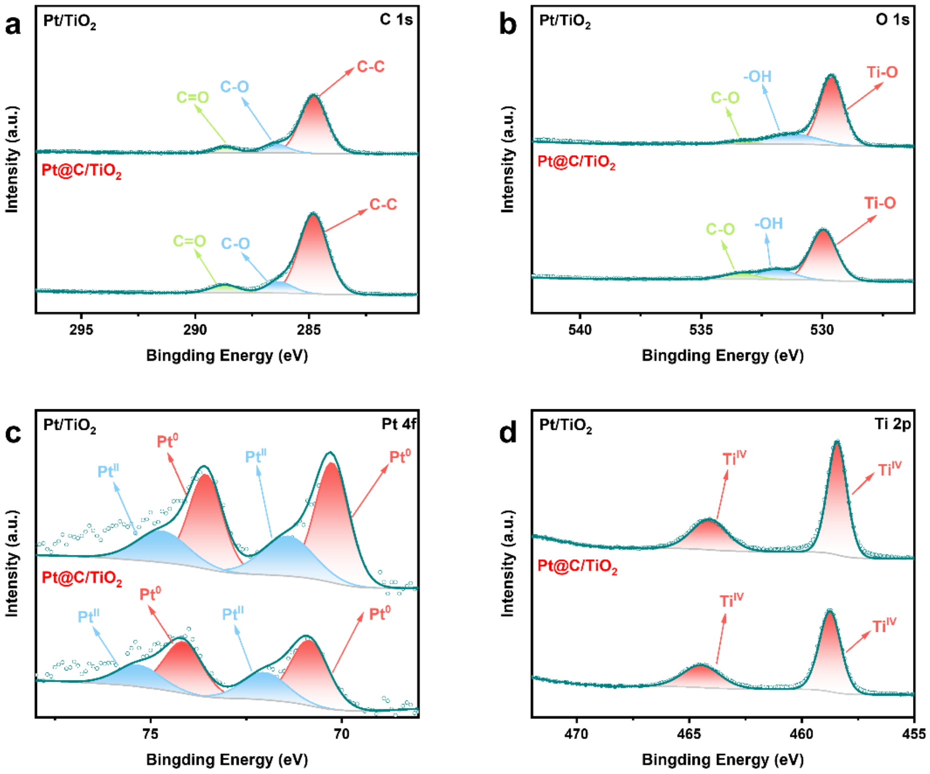

Figure S4. XPS peak fitting results of Pt/TiO2 and Pt@C/TiO_2. (a) XPS core-level spectra of C 1s; (b) XPS core-level spectra of O 1s; (c) XPS core-level spectra of Pt 4f; (d) XPS core-level spectra of Ti 2p.

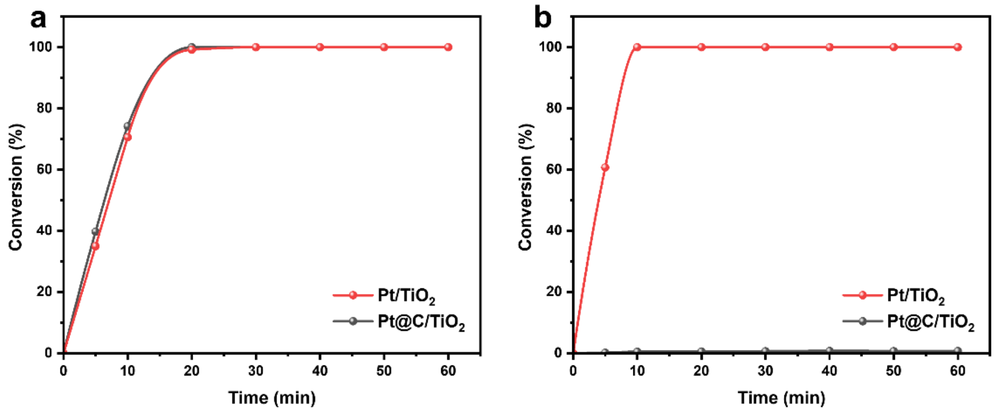

Figure S5. Time-dependent catalysis of nitrobenzene(a) or styrene(b) by Pt/TiO_2 and Pt@C/TiO_2. Reaction conditions: 60 ºC, 1 bar H_2, 0.001 mol of Pt/mol of each substrate, 10 mL ethanol as solvent.

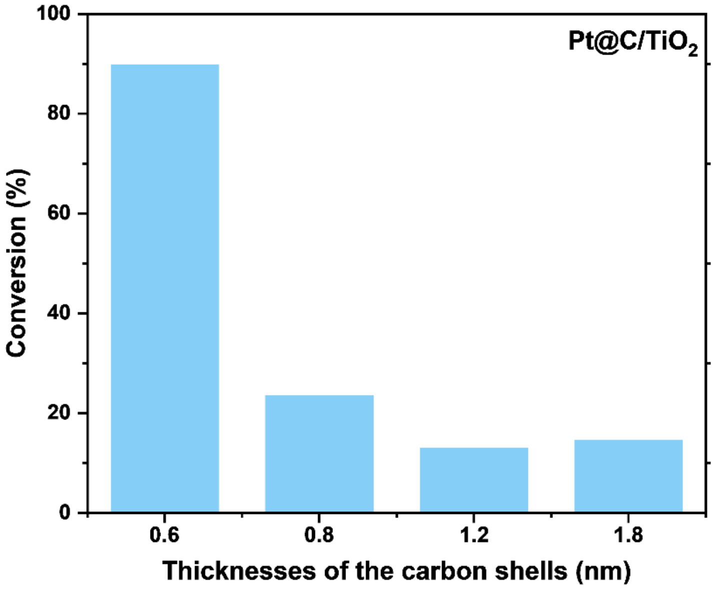

Figure S6. Conversion of 4-nitrostyrene by the catalysts with different carbon shell thicknesses. Reaction conditions: 60 ^{o}C, 1 bar H_2, 0.001 mol of Pt/mol of each substrate, 10 mL ethanol as solvent.

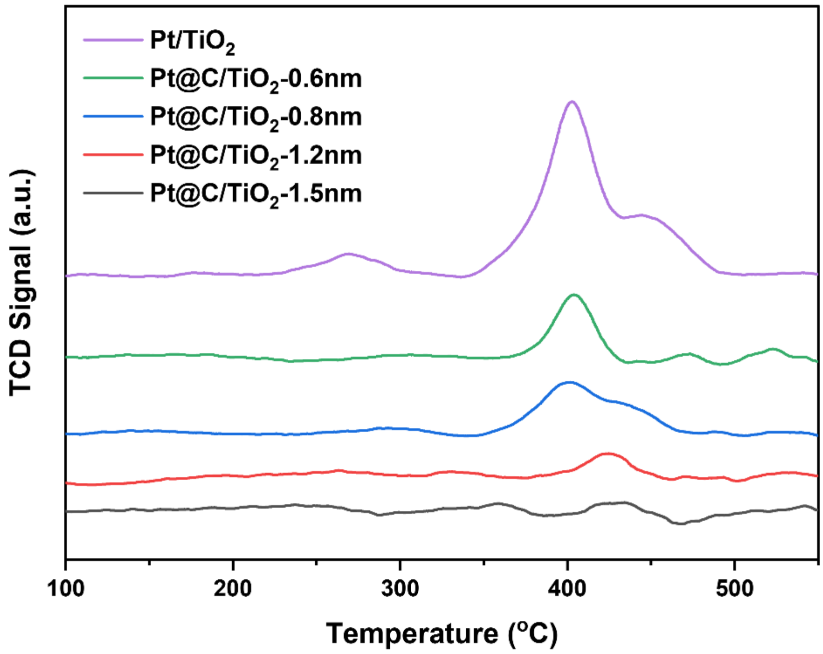

Figure S7. H_2-TPD curves for Pt/TiO_2, Pt@C/TiO_2-0.6nm, Pt@C/TiO_2-0.8nm, Pt@C/TiO_2-1.2nm and Pt@C/TiO_2-1.5nm.

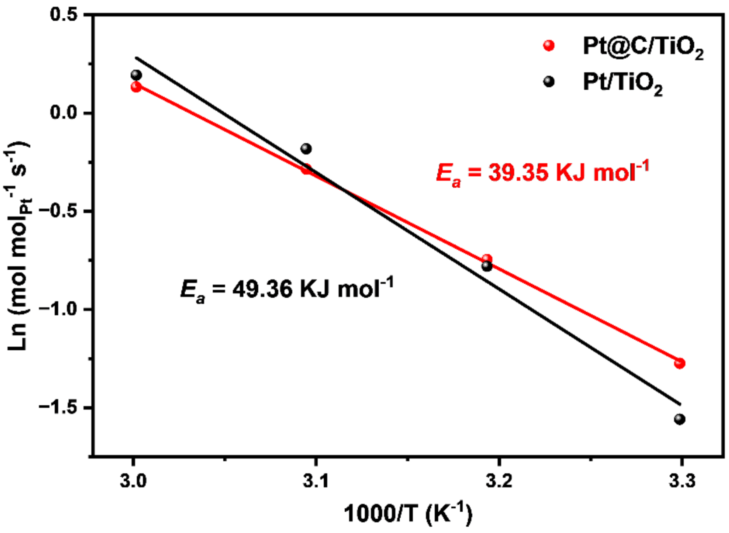

Figure S8. The *E*a of -NO_2 group hydrogenation over the Pt/TiO_2 and Pt@C/TiO_2.

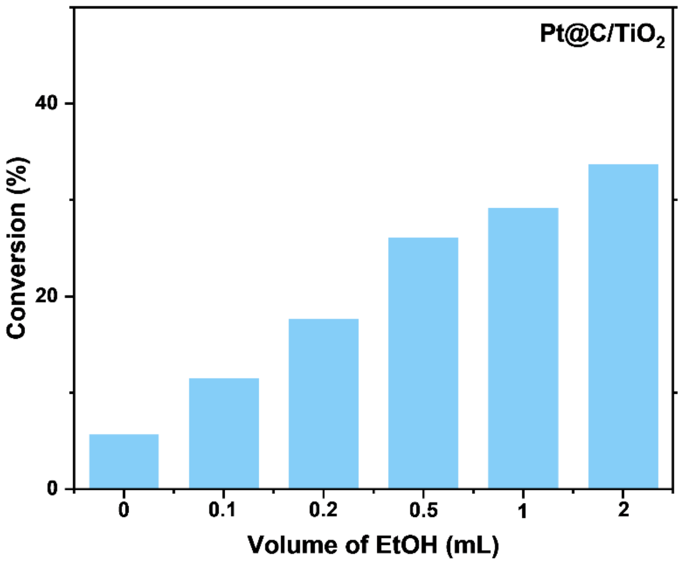

Figure S9. Conversion of 4-nitrostyrene over the Pt@C/TiO_2. Reaction conditions: 60 ºC, 1 bar H_2, 1 mmol of substrate, 19.5 mg of samples and 10 mL mixture solvent of THF and EtOH.

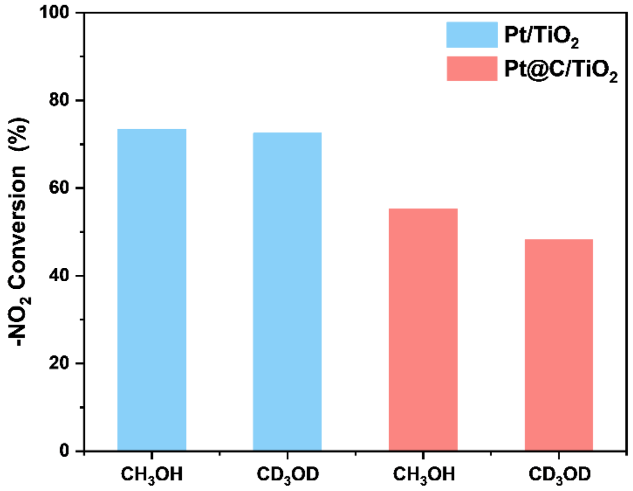

Figure S10. Conversion of -NO_2 group over the Pt/TiO_2 and Pt@C/TiO_2 at 20 min. Reaction conditions: 60 ^{o}C, 1 bar H_2, 0.001 mol of Pt/mol of each substrate, 10 mL ethanol as solvent.

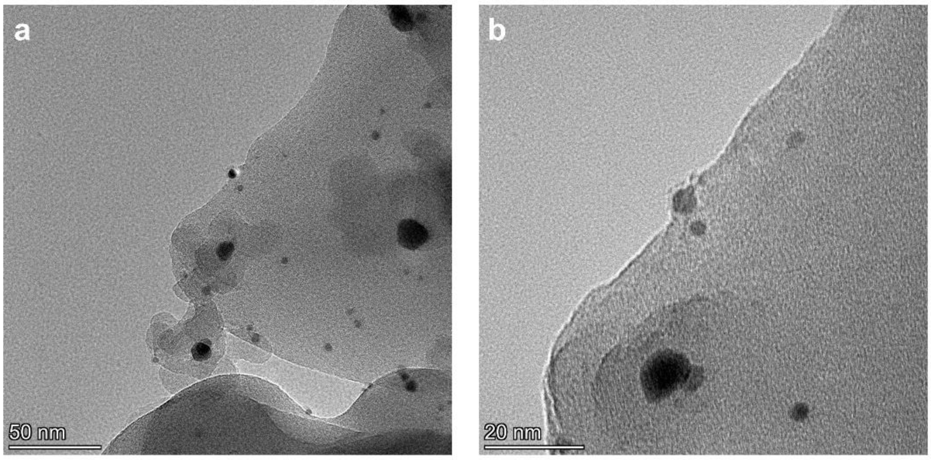

Figure S11. TEM images of the Pt@C/SiO_2

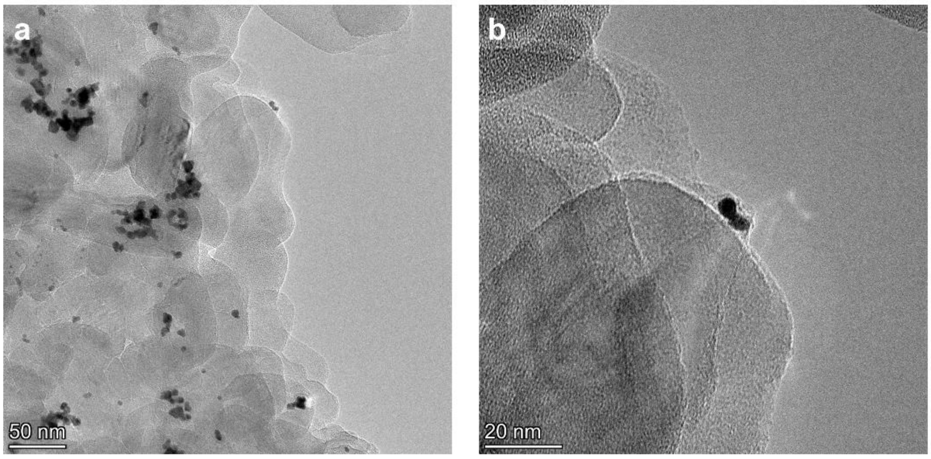

Figure S12. TEM images of the Pt@C/BN.

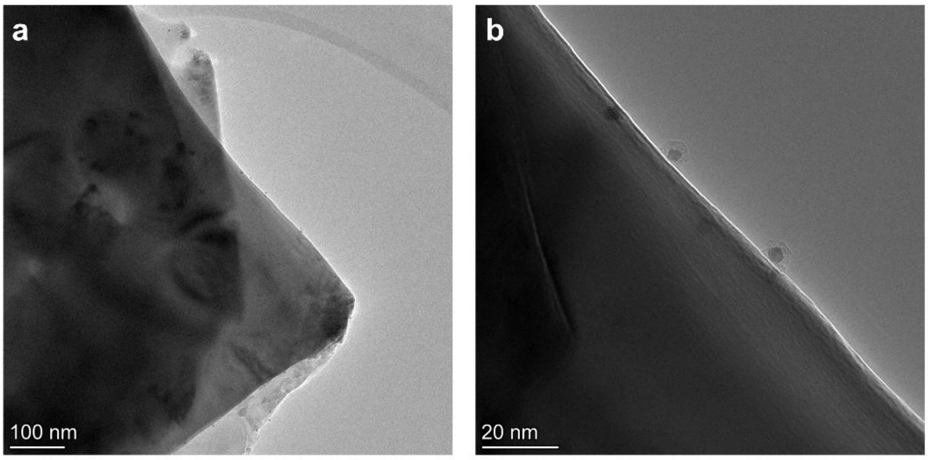

Figure S13. TEM images of the $Pt@C/Al_2O_3$.

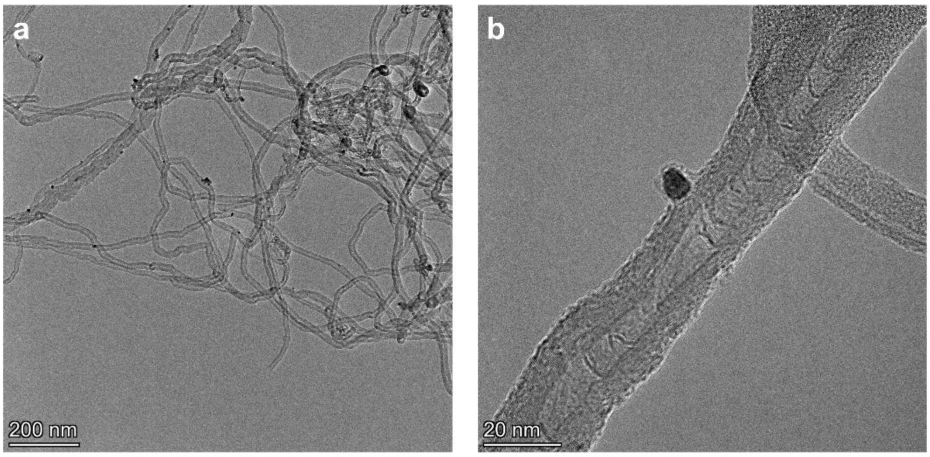

Figure S14. TEM images of the Pt@C/CNT.

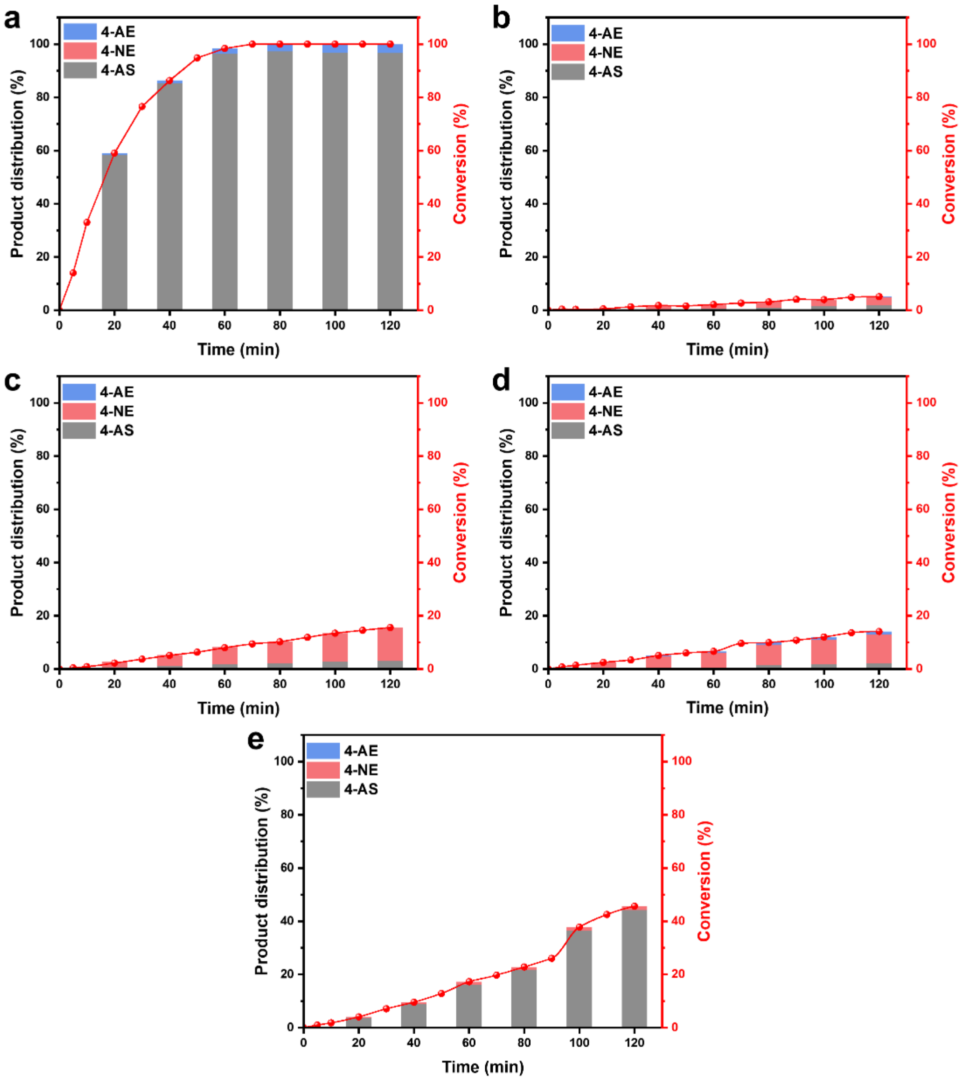

Figure S15. Time-dependent catalysis of 4-nitrostyrene by Pt@C/TiO_2(a), Pt@C/SiO_2(b), Pt@C/BN(c), Pt@C/Al_2O_3(d) and Pt@C/CNT(e). Reaction conditions: 60 ºC, 1 bar H_2, 0.001 mol of Pt/mol of each substrate, 10 mL ethanol as solvent.

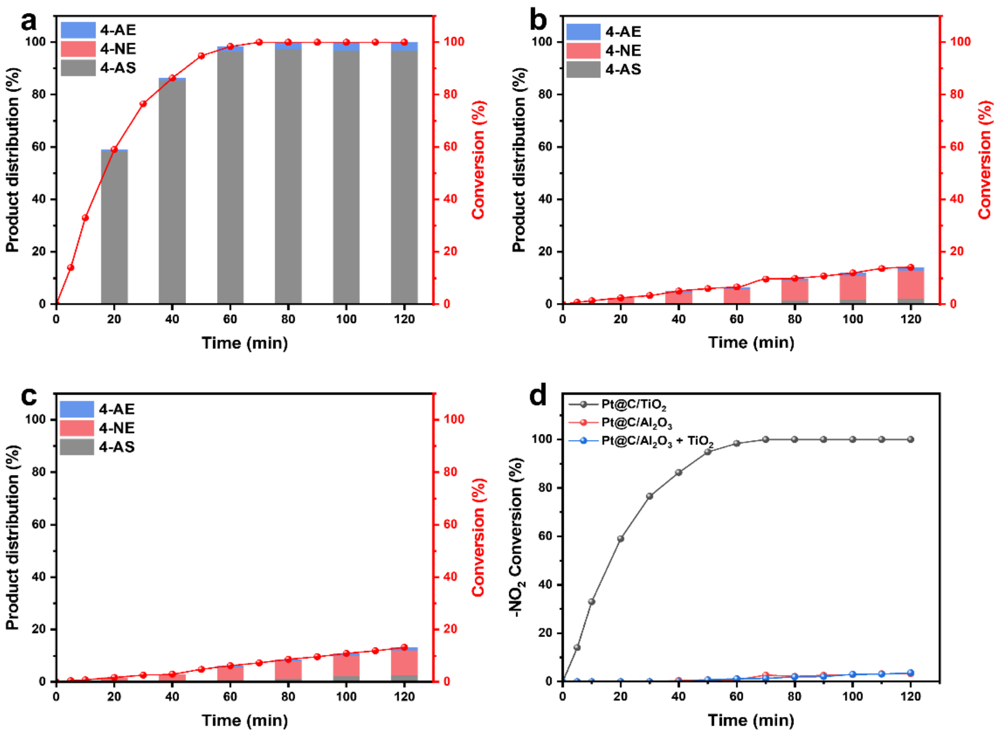

Figure S16. Time-dependent catalysis of 4-nitrostyrene by Pt@C/TiO_2 (a), Pt@C/Al_2O_3 (b), Pt@C/Al_2O_3 + TiO_2 (c); (d) Time-dependent catalysis of –NO_2. Reaction conditions: 60 ºC, 1 bar H_2, 0.001 mol of Pt/mol of each substrate, 10 mL ethanol as solvent.

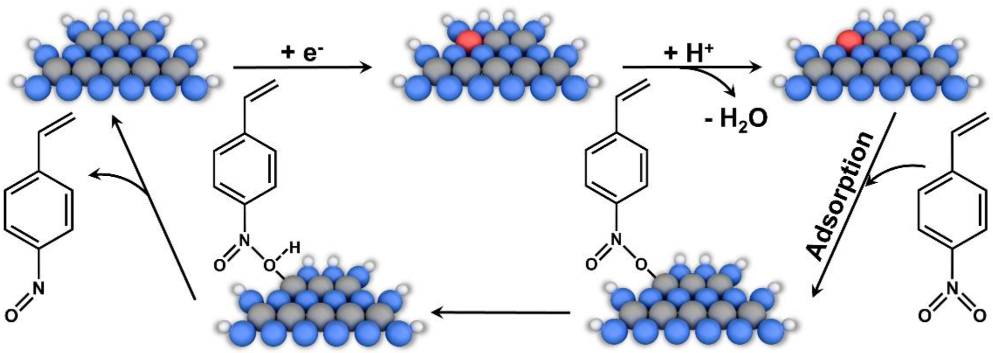

Figure S17. Proposed mechanism of catalytic deoxygenation of -NO_2 group on TiO_2.

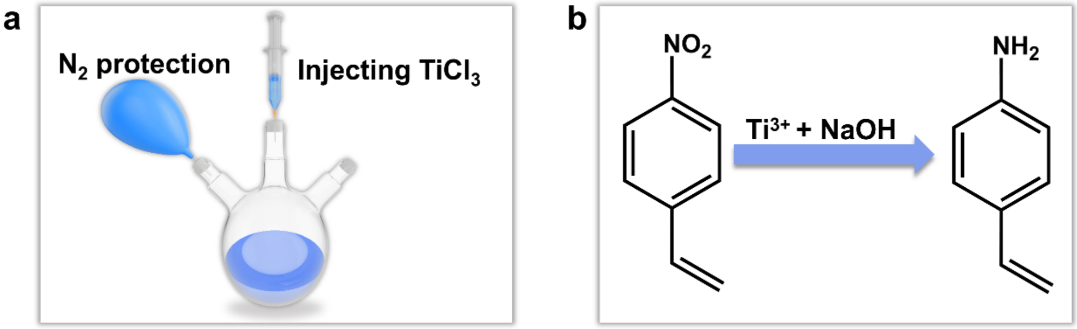

Figure S18. (a) Schematic diagram of the reaction device; (b) Scheme of hydrogenation of 4-NS by $TiCl_3$ with NaOH;

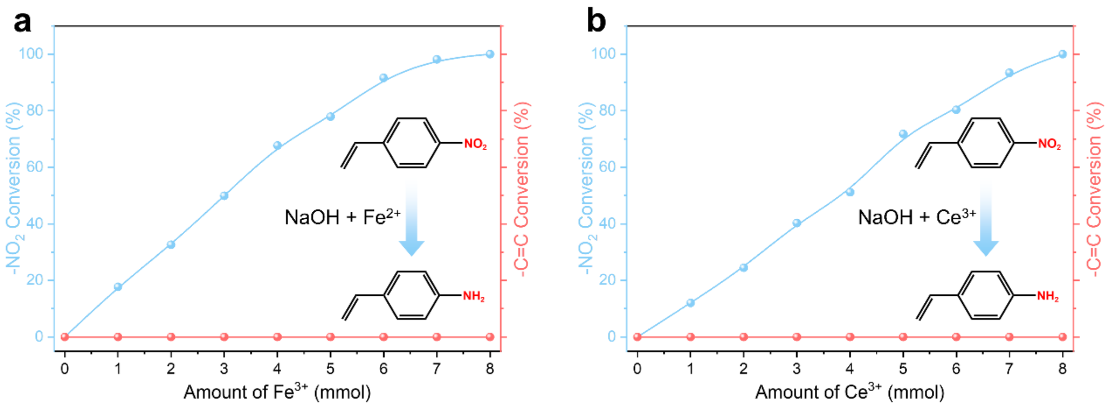

Figure S19. (a) Conversion of -NO_2 and C=C group as a function of Fe^{2+} consumption; (b) Conversion of -NO_2 and C=C group as a function of Ce^{3+} consumption;

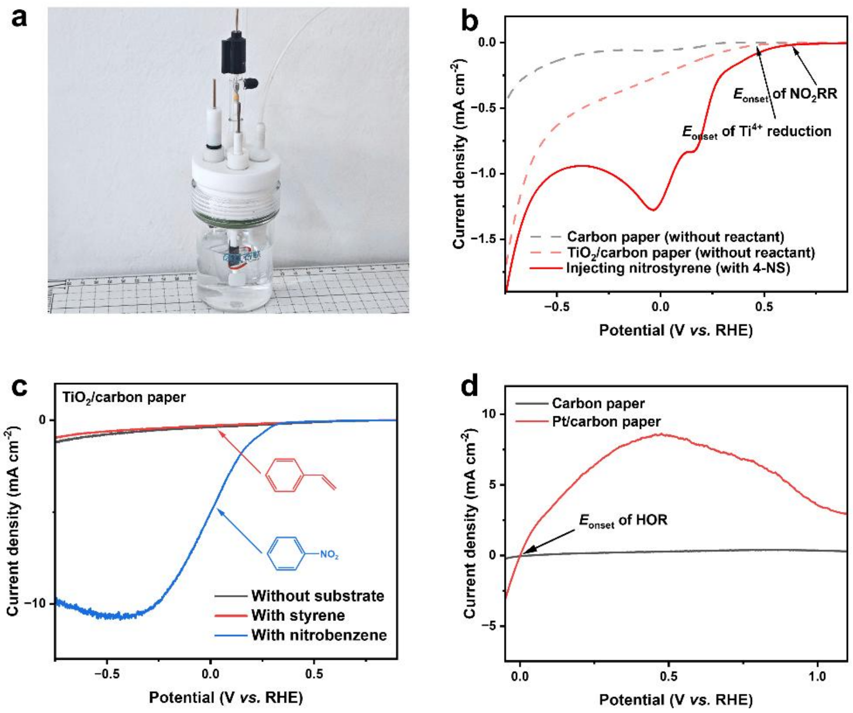

Figure S20. Half-cell electrochemical experiments for $–NO_2$ reduction or H_2 oxidation. (a) Photograph of half-cell electrochemical experiments; (b) LSV curves recorded on TiO_2/carbon paper electrode or carbon paper; (c) LSV curves recorded on TiO_2/carbon paper electrode. (d) LSV curves of HOR over clean carbon paper electrode and Pt/carbon paper electrode. Reaction conditions: 0.1 M KOH as electrolyte, 25 ^{o}C, N_2 flow for (b) and (c), H_2 flow for (d), scan rate 10 mV s^{-1}.

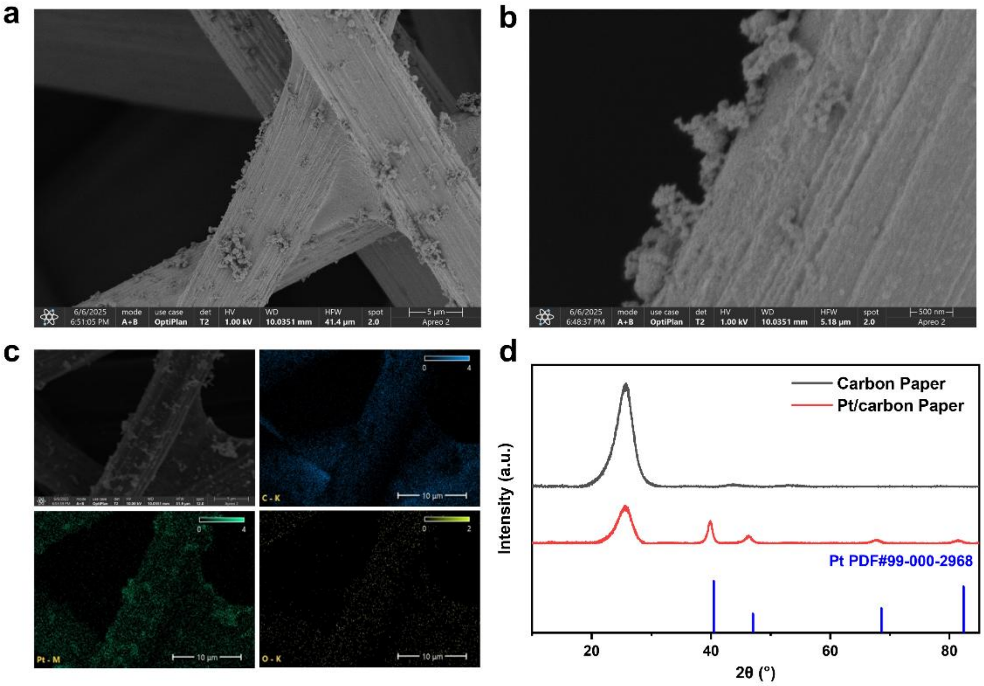

Figure S21. The morphology and crystal structure of Pt/carbon paper. (a and b) SEM images, (c) EDS mapping and (d) XRD of the Pt/carbon paper.

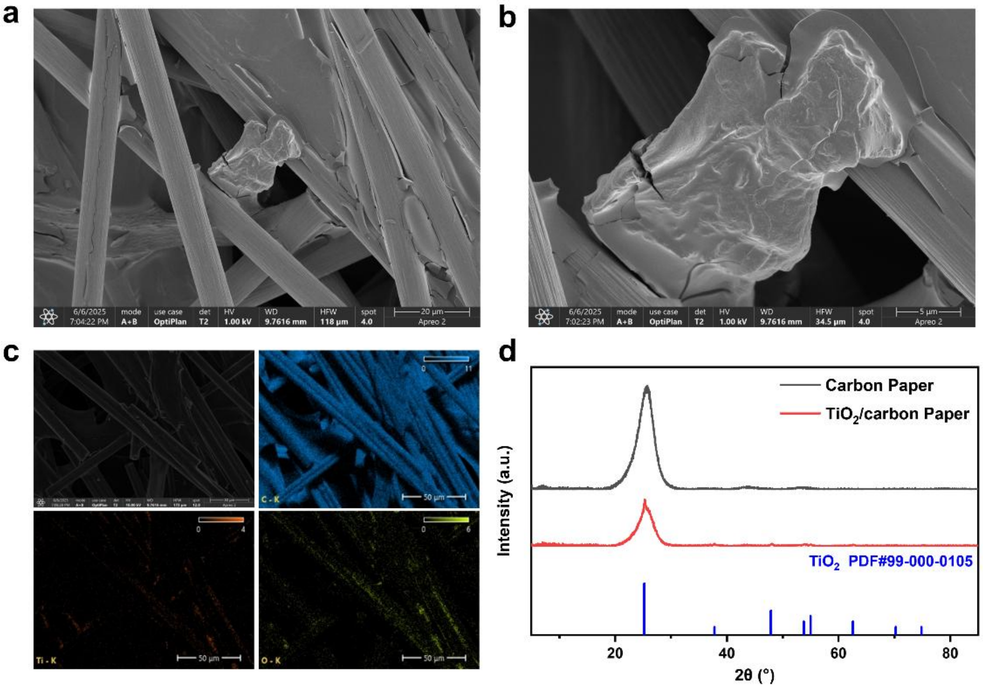

Figure S22. The morphology and crystal structure of TiO_2/carbon paper. (a and b) SEM images, (c) EDS mapping and (d) XRD of the TiO_2/carbon paper.

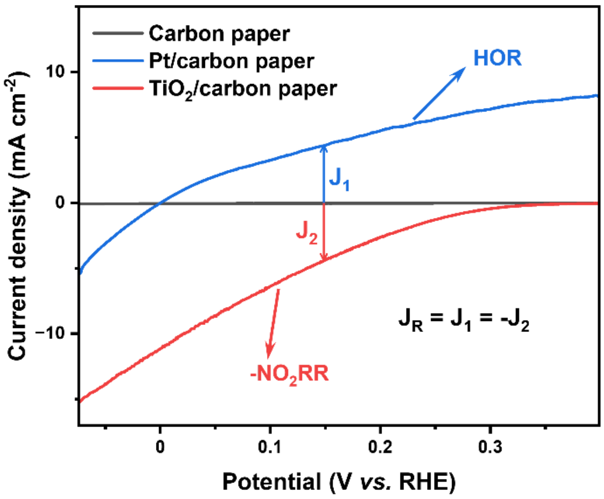

Figure S23 Overlay of the polarization curves for the hydrogen oxidation reaction (HOR) on Pt and the nitro-group reduction reaction (–NO_2RR) on TiO_2.

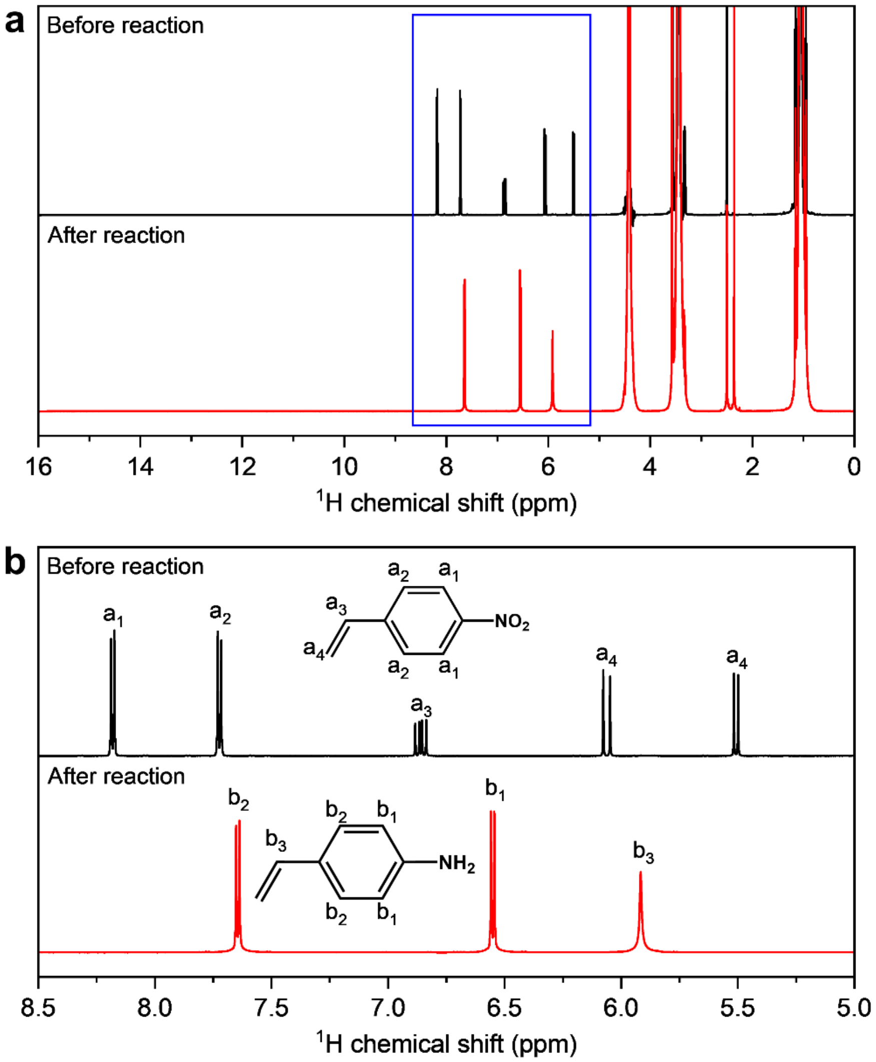

Figure S24. Hydrogenation of 4-nitrostyrene over Pt@C-TiO_2. (a) ^{1}H-NMR spectra referenced to the residual proton signal of incompletely deuterated DMSO-d6. (b) Enlarged view of the blue rectangular area showing the ^{1}H-NMR information of hydrogen. Black and red curves represent the reactants and the products, respectively. After separating the catalyst by centrifugation and filtration, the products were subjected to NMR analysis without any further purification. Reaction conditions: 60 °C, 1 bar H_2, 0.001 mol of Pt/mol of each substrate, 10 mL ethanol as solvent.

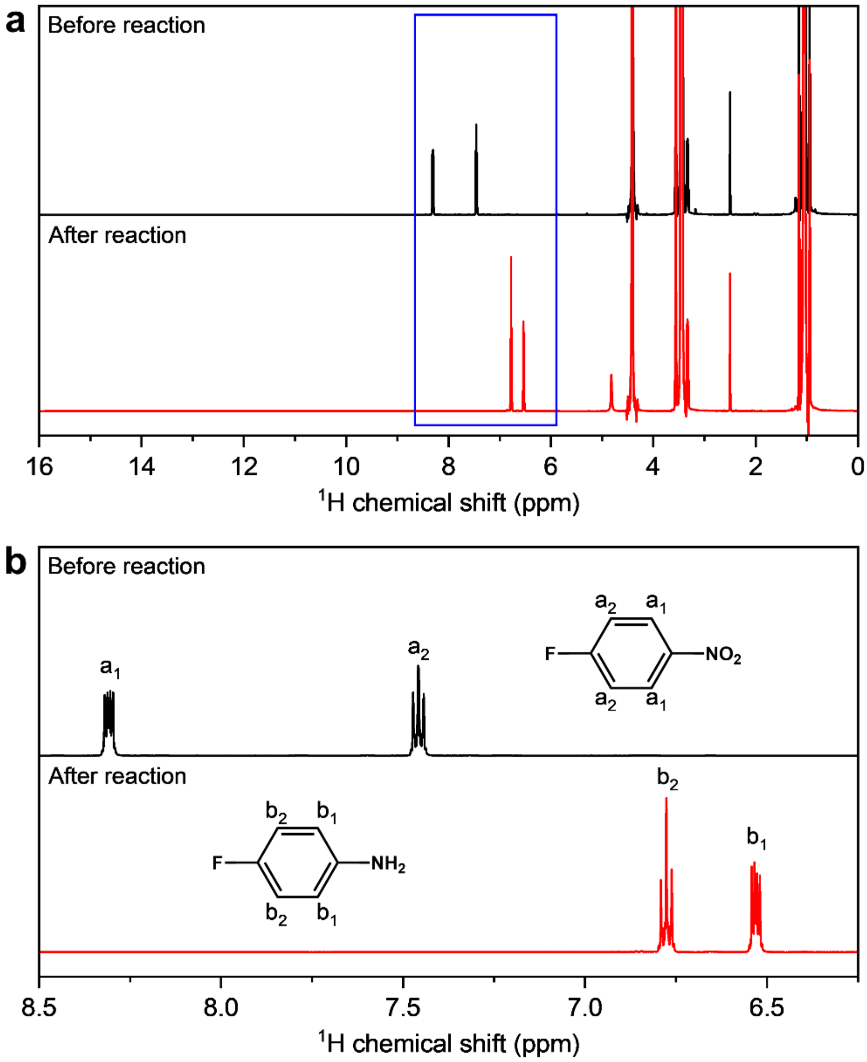

Figure S25. Hydrogenation of 4-fluoronitrobenzene over Pt@C-TiO_2. (a) ^{1}H-NMR spectra referenced to the residual proton signal of incompletely deuterated DMSO-d6. (b) Enlarged view of the blue rectangular area showing the ^{1}H-NMR information of hydrogen. Black and red curves represent the reactants and the products, respectively. After separating the catalyst by centrifugation and filtration, the products were subjected to NMR analysis without any further purification. Reaction conditions: 60 ^oC, 1 bar H_2, 0.001 mol of Pt/mol of each substrate, 10 mL ethanol as solvent.

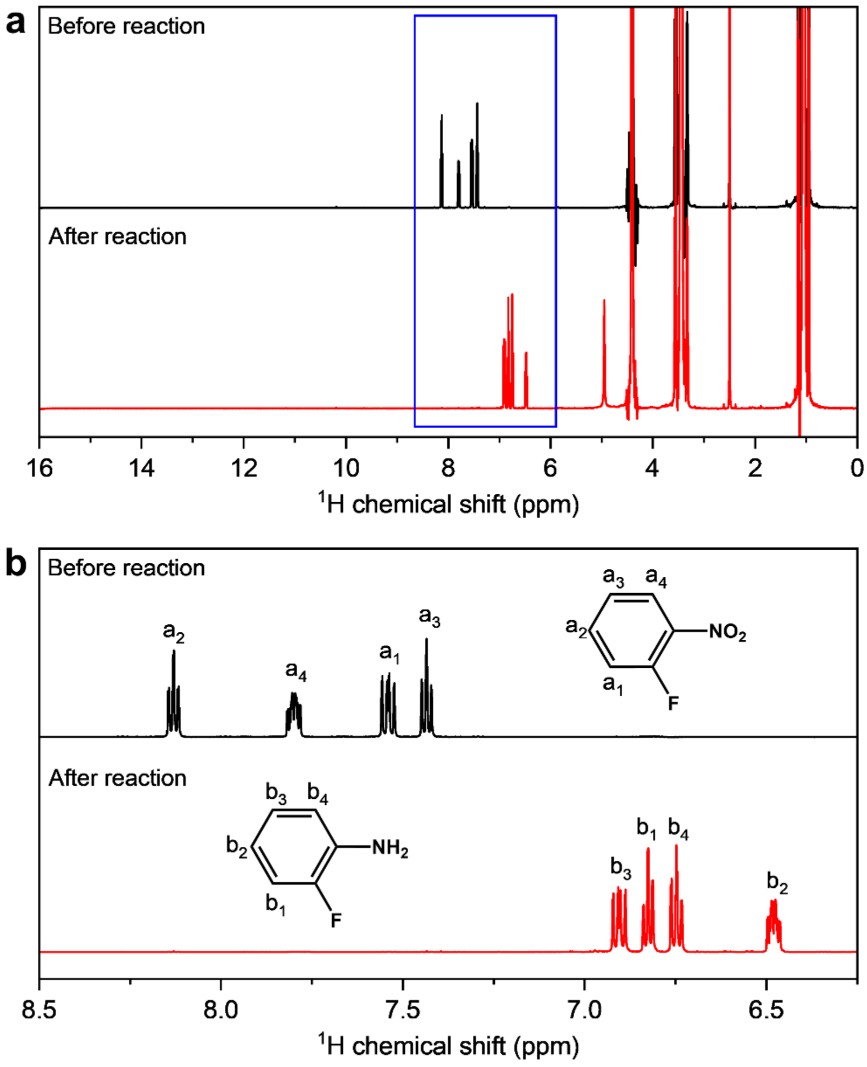

Figure S26. Hydrogenation of 2-fluoronitrobenzene over Pt@C-TiO_2. (a) ^{1}H-NMR spectra referenced to the residual proton signal of incompletely deuterated DMSO-d6. (b) Enlarged view of the blue rectangular area showing the ^{1}H-NMR information of hydrogen. Black and red curves represent the reactants and the products, respectively. After separating the catalyst by centrifugation and filtration, the products were subjected to NMR analysis without any further purification. Reaction conditions: 60 °C, 1 bar H_2, 0.001 mol of Pt/mol of each substrate, 10 mL ethanol as solvent.

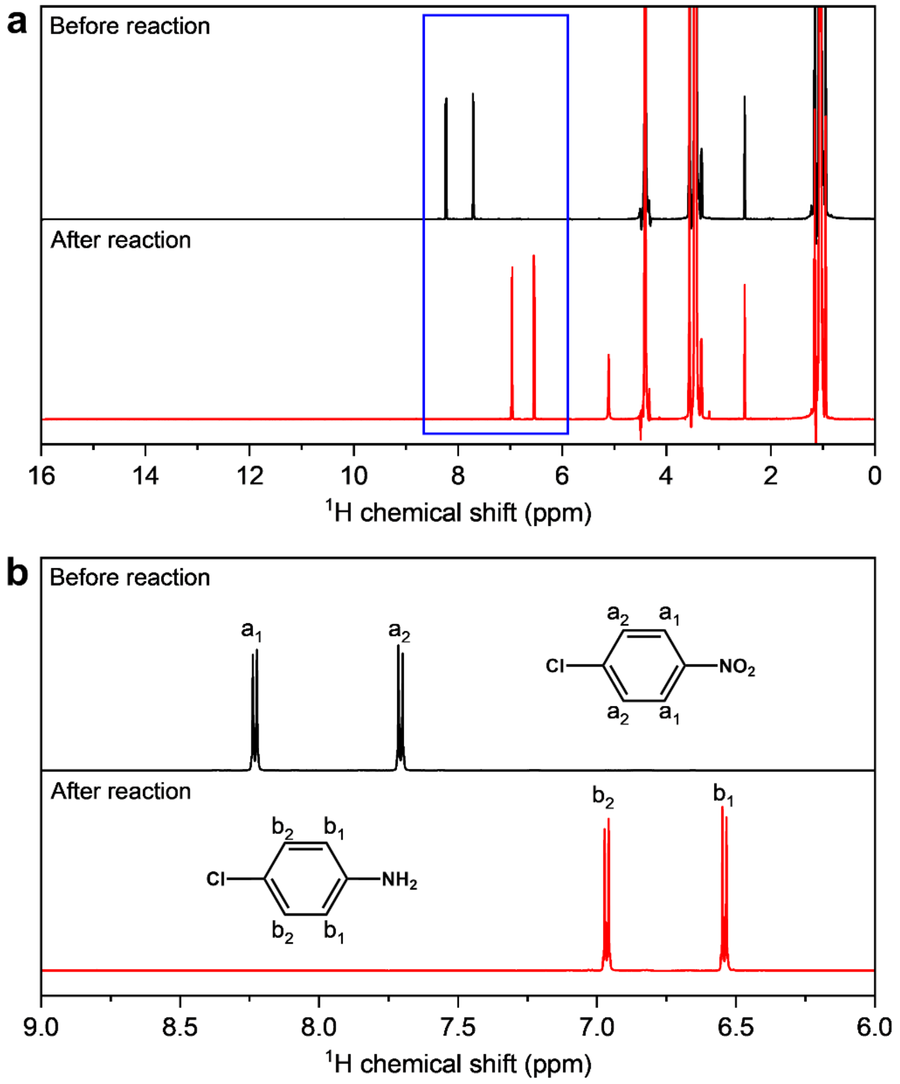

Figure S27. Hydrogenation of 4-nitrochlorobenzene over Pt@C-TiO_2. (a) ^{1}H-NMR spectra referenced to the residual proton signal of incompletely deuterated DMSO-d6. (b) Enlarged view of the blue rectangular area showing the ^{1}H-NMR information of hydrogen. Black and red curves represent the reactants and the products, respectively. After separating the catalyst by centrifugation and filtration, the products were subjected to NMR analysis without any further purification. Reaction conditions: 60 ^{o}C, 1 bar H_2, 0.001 mol of Pt/mol of each substrate, 10 mL ethanol as solvent.

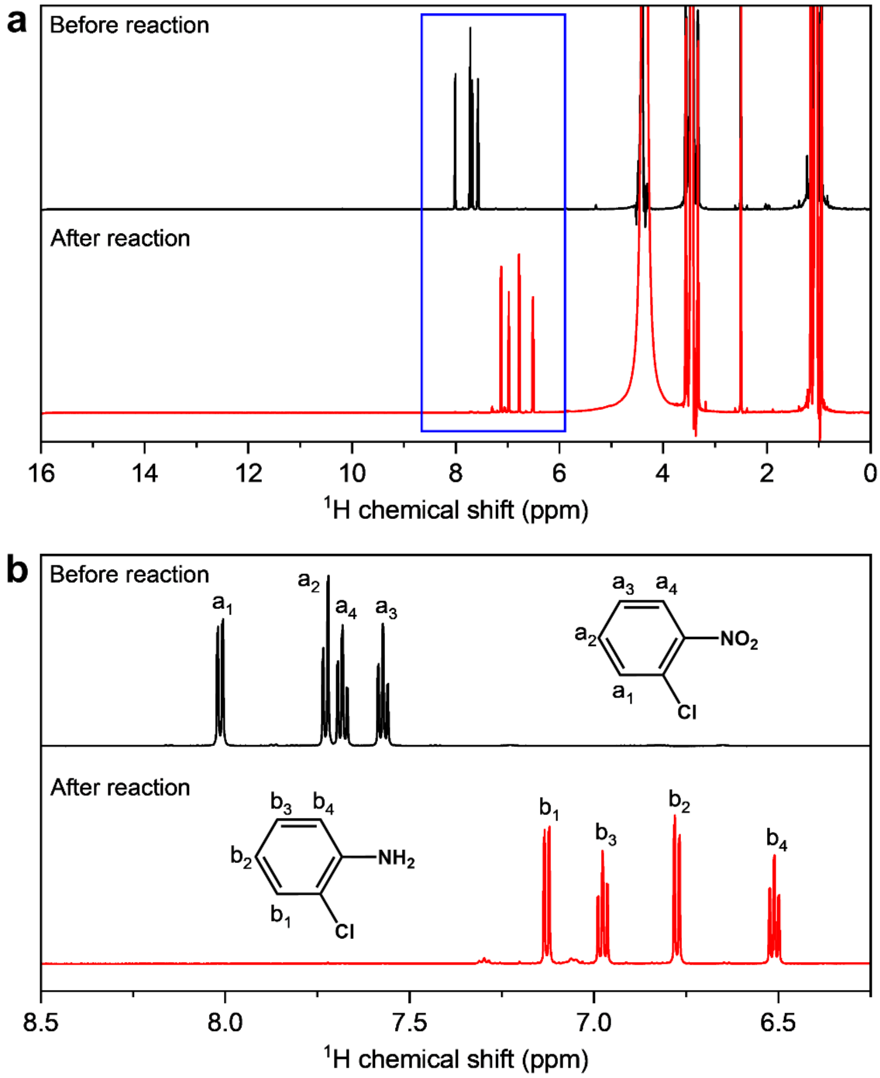

Figure S28. Hydrogenation of 2-nitrochlorobenzene over Pt@C-TiO_2. (a) ^{1}H-NMR spectra referenced to the residual proton signal of incompletely deuterated DMSO-d6. (b) Enlarged view of the blue rectangular area showing the ^{1}H-NMR information of hydrogen. Black and red curves represent the reactants and the products, respectively. After separating the catalyst by centrifugation and filtration, the products were subjected to NMR analysis without any further purification. Reaction conditions: 60 °C, 1 bar H_2, 0.001 mol of Pt/mol of each substrate, 10 mL ethanol as solvent.

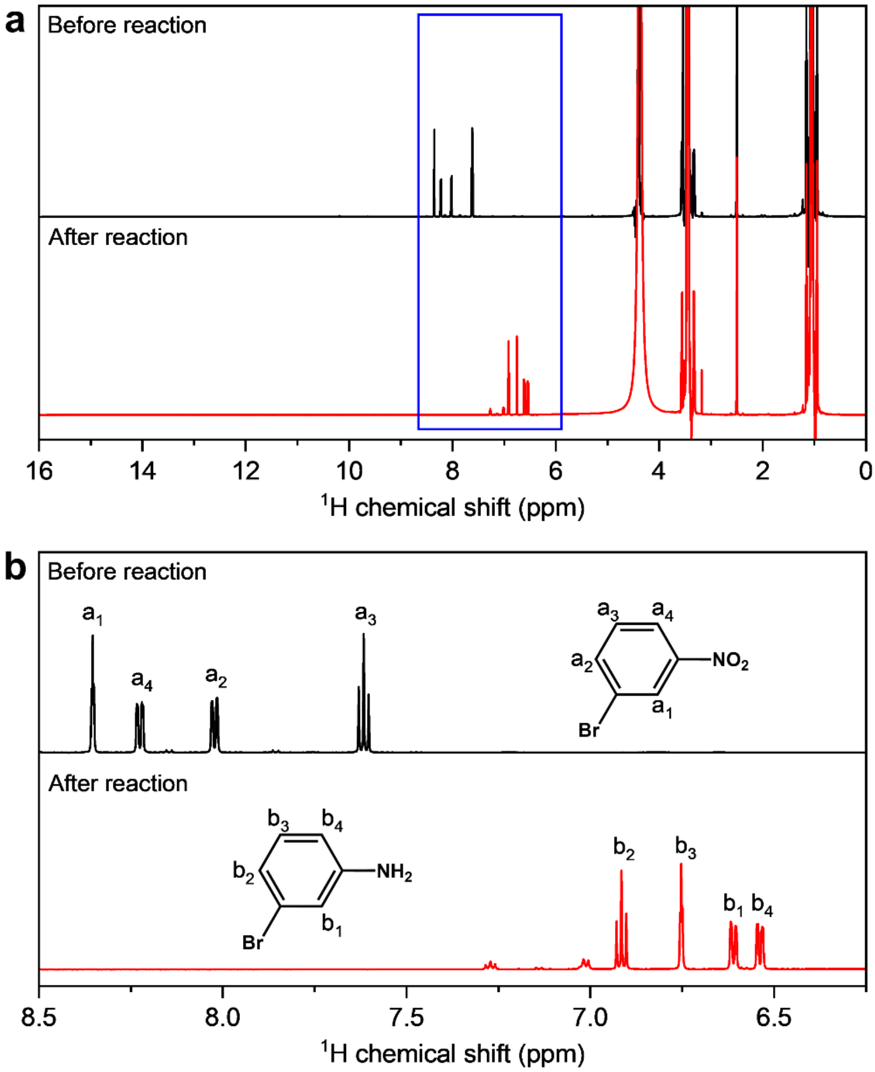

Figure S29. Hydrogenation of 3-bromonitrobenzene over Pt@C-TiO_2. (a) ^{1}H-NMR spectra referenced to the residual proton signal of incompletely deuterated DMSO-d6. (b) Enlarged view of the blue rectangular area showing the ^{1}H-NMR information of hydrogen. Black and red curves represent the reactants and the products, respectively. After separating the catalyst by centrifugation and filtration, the products were subjected to NMR analysis without any further purification. Reaction conditions: 60 ^oC, 1 bar H_2, 0.001 mol of Pt/mol of each substrate, 10 mL ethanol as solvent.

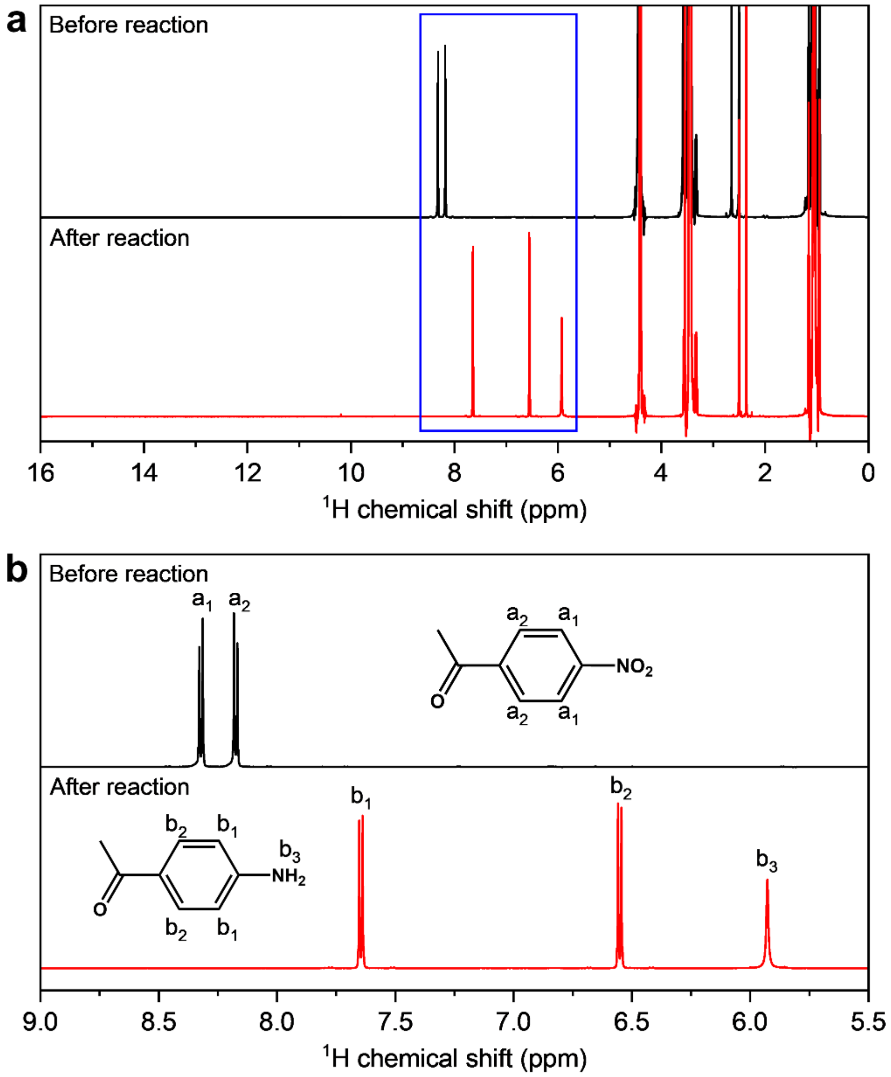

Figure S30. Hydrogenation of 4-nitroacetophenone over Pt@C-TiO_2. (a) ^{1}H-NMR spectra referenced to the residual proton signal of incompletely deuterated DMSO-d6. (b) Enlarged view of the blue rectangular area showing the ^{1}H-NMR information of hydrogen. Black and red curves represent the reactants and the products, respectively. After separating the catalyst by centrifugation and filtration, the products were subjected to NMR analysis without any further purification. Reaction conditions: 60 ^{o}C, 1 bar H_2, 0.001 mol of Pt/mol of each substrate, 10 mL ethanol as solvent.

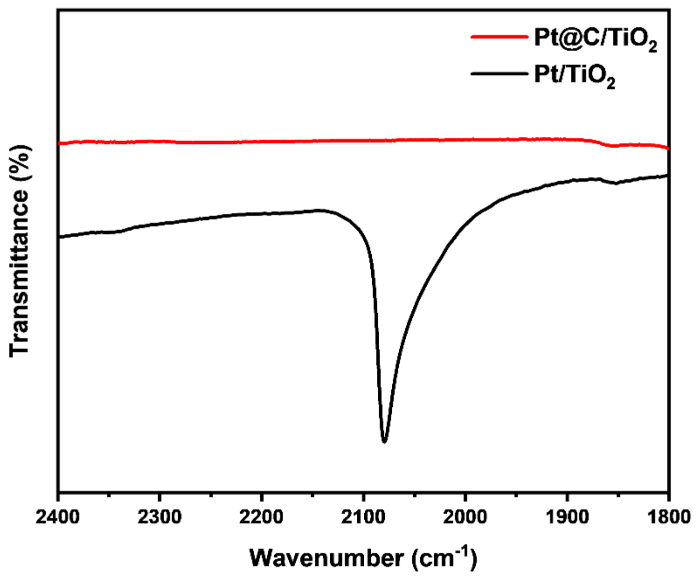

Figure S31. FTIR of adsorption of CO on the Pt/TiO_2 or Pt@C/TiO_2.

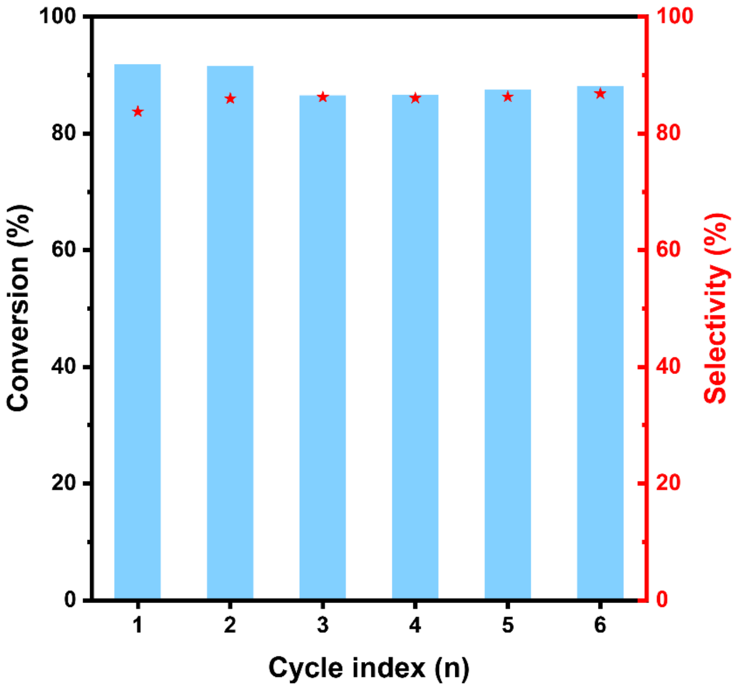

Figure S32. The recycling stability of Pt@C/TiO_2 in the hydrogenation of 4-nitrostyrene. Reaction conditions: 60 °C, 1 bar H_2, 1 mmol of substrate, 19.5 mg of samples and 10 mL ethanol as solvent.

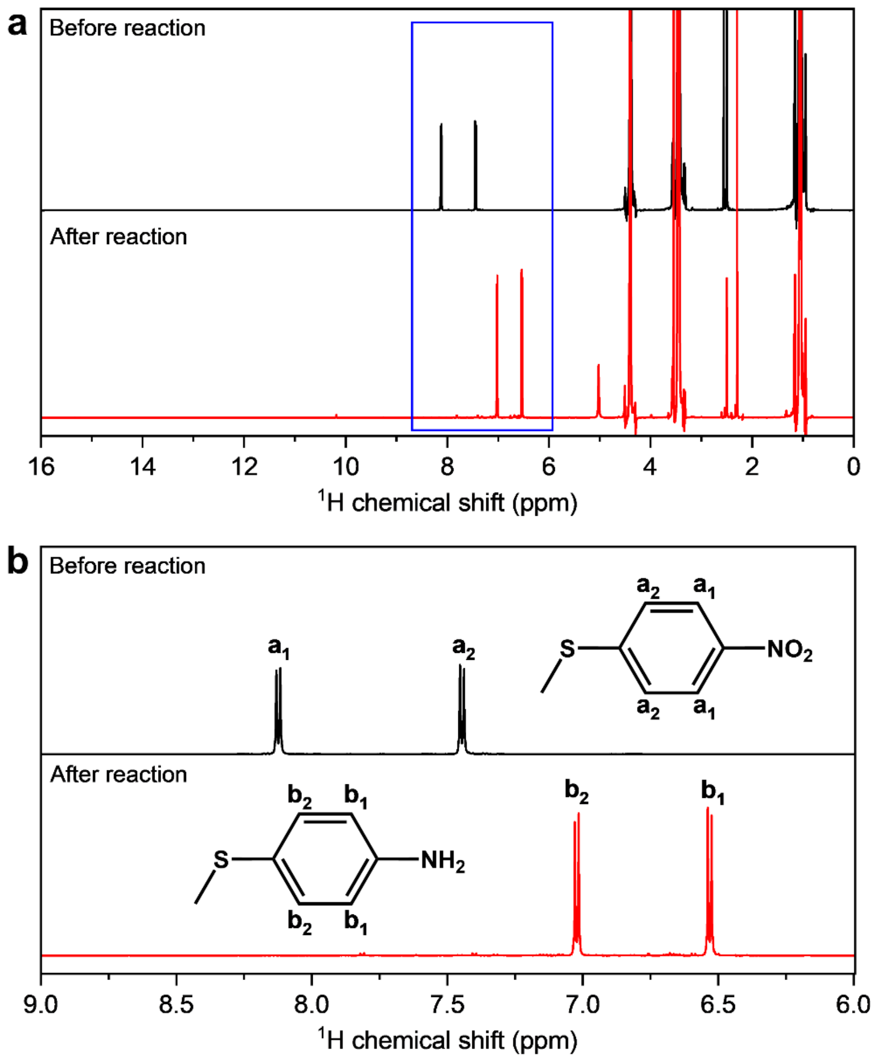

Figure S33. Hydrogenation of 4-nitrobenzyl methyl sulfide over Pt@C-TiO_2. (a) ^{1}H-NMR spectra referenced to the residual proton signal of incompletely deuterated DMSO-d6. (b) Enlarged view of the blue rectangular area showing the ^{1}H-NMR information of hydrogen. Black and red curves represent the reactants and the products, respectively. After separating the catalyst by centrifugation and filtration, the products were subjected to NMR analysis without any further purification. Reaction conditions: 60 ^oC, 3 bar H_2, 0.002 mol of Pt/mol of each substrate, 10 mL ethanol as solvent.

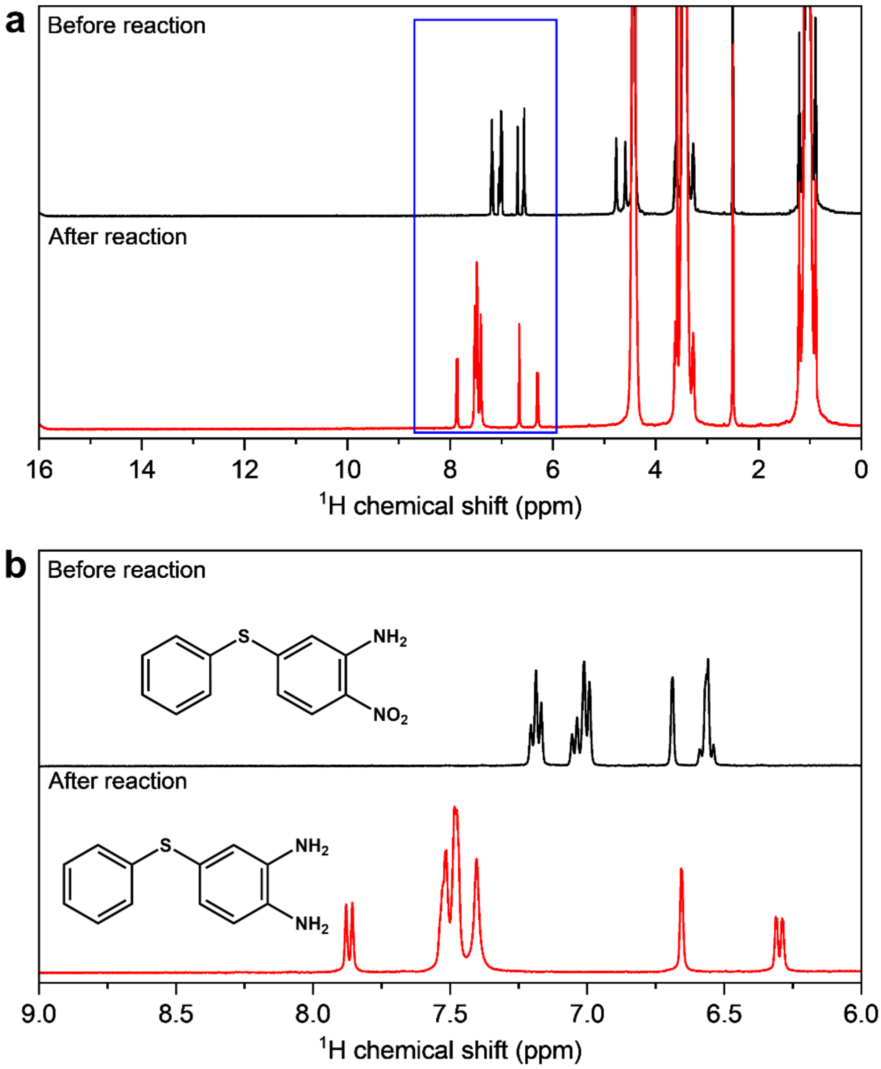

Figure S34. Hydrogenation of 2-nitro-5-(phenylthio)aniline over Pt@C-TiO_2. (a) ^{1}H-NMR spectra referenced to the residual proton signal of incompletely deuterated DMSO-d6. (b) Enlarged view of the blue rectangular area showing the ^{1}H-NMR information of hydrogen. Black and red curves represent the reactants and the products, respectively. After separating the catalyst by centrifugation and filtration, the products were subjected to NMR analysis without any further purification. Reaction conditions: 60 °C, 3 bar H_2, 0.002 mol of Pt/mol of each substrate, 10 mL ethanol as solvent.

Table S1. Catalytic hydrogenation of nitroaromatics with different substituents.

Entry	Reactant	Product	Pt@C/TiO_2		Pt/C	
			Conv. (%)	Sel. (%)	Conv. (%)	Sel. (%)
1	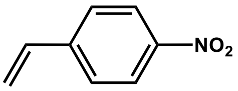	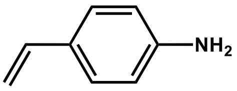	98.39	97.93	~100	0
2	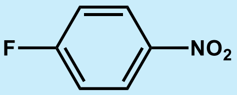	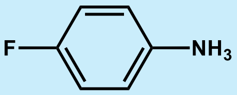	~100	~100	~100	97.91
3	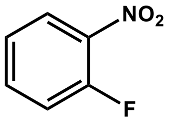	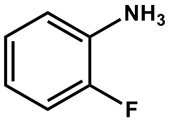	~100	~100	~100	95.64
4	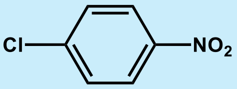	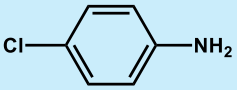	~100	~100	~100	41.59
5	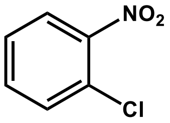	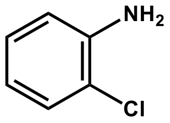	~100	98.23	~100	31.63
6	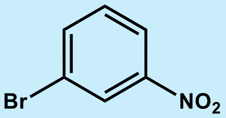	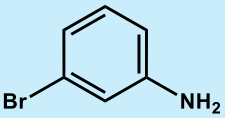	~100	95.68	~100	24.51
7	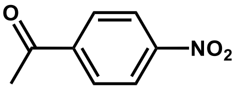	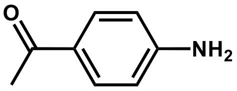	~100	~100	~100	84.95

Table S2. Pt content of samples based on ICP–OES spectra.

Simplified name in paper	Pt content (wt%)
Pt/TiO_2	1.28
Pt@C/TiO_2	1.20
Pt@C/TiO_2-0.5ml	1.20
Pt@C/TiO_2-1ml	0.93
Pt/TiO_2-3ml	0.88
Pt/TiO_2-5ml	0.81
Pt/Al_2O_3	0.96
Pt/BN	1.12
Pt/CNT	1.00
Pt/SiO_2	1.03

Table S3. Comparison of the activity and selectivity of Pt, Pd, Ru or Ir-based catalysts in the literature for nitro-styrene hydrogenation into aminostyrene.

Catalyst	Reaction condition	Time (min)	Conv. (%)	Sel. (%)	Ref.
Pt@C/TiO_2	S/Pt (1000), 60 °C, 1 bar H_2, ethanol	60	99.0	97.9	This work
Pt/TiO_2	S/Pt (1000), 60 °C, 1 bar H_2, ethanol	30	~100	15.6	This work
0.4% RuNi SAA	S/Ru (1263), 60 °C, 10 bar H_2, ethanol	180	~100	>99	S1
Pt@Fe_2O_3-HV_O	S/ Pt (634), 35 °C, 10bar H_2, toluene	60	87.9	99.3	S2
Ir_1Ni/Al_2O_3	S/ Ir (1068), 50 °C, 10bar H_2, ethanol	240	>98	>98	S3
0.2 wt% Pt/TiO_2	S/Pt (323), 40 °C, 3 bar H_2, toluene	390	95	93.1	S4
Ir_1Mo_1/TiO_2	S/Ir (325), 120 °C, 20 bar H_2, toluene	60	100	96	S5
$Fe(OH)_x$/Pt	S/Pt (1000), 60 °C, 1 bar H_2, ethanol	120	~100	92.8	S6
Pd/Ti_3SiC_2	S/Pd (1315), 140 °C, 25 bar H_2, heptane	1440	100	93	S7
0.25%Pt_1/MoC	S/Pt (3152), 70 °C, 20 bar H2, cyclohexane	90	100	88	S8

References

(1) Liu, W.; Feng, H.; Yang, Y.; Niu, Y.; Wang, L.; Yin, P.; Hong, S.; Zhang, B.; Zhang, X.; Wei, M. 1-Highly-Efficient RuNi Single-Atom Alloy Catalysts toward Chemoselective Hydrogenation of Nitroarenes. *Nat. Commun.* **2022**, *13* (1), 3188. https://doi.org/10.1038/s41467-022-30536-9.

(2) Yue, G.; Yu, Y.; Li, S.; Li, H.; Gao, S.; Wang, Y.; Guo, W.; Wang, N.; Li, X.; Cui, Z.; Cao, C.; Jiang, L.; Zhao, Y. 2-Boosting Chemoselective Hydrogenation of Nitroaromatic via Synergy of Hydrogen Spillover and Preferential Adsorption on Magnetically Recoverable pt@Fe_2O_3. *Small* **2023**, *19* (11), 2207918. https://doi.org/10.1002/smll.202207918.

(3) Feng, H.; Liu, W.; Wang, L.; Xu, E.; Pang, D.; Ren, Z.; Wang, S.; Zhao, S.; Deng, Y.; Liu, T.; Yang, Y.; Zhang, X.; Li, F.; Wei, M. 3-Rational Design and Precise Synthesis of Single-atom Alloy Catalysts for the Selective Hydrogenation of Nitroarenes. *Adv. Sci.* **2024**, *11* (23), 2304908. https://doi.org/10.1002/advs.202304908.

(4) Corma, A.; Serna, P.; Concepción, P.; Calvino, J. J. 4-Transforming Nonselective into Chemoselective Metal Catalysts for the Hydrogenation of Substituted Nitroaromatics. *J. Am. Chem. Soc.* **2008**, *130* (27), 8748–8753. https://doi.org/10.1021/ja800959g.

(5) Fu, J.; Dong, J.; Si, R.; Sun, K.; Zhang, J.; Li, M.; Yu, N.; Zhang, B.; Humphrey, M. G.; Fu, Q.; Huang, J. 5-Synergistic Effects for Enhanced Catalysis in a Dual Single-Atom Catalyst. *ACS Catal.* **2021**, *11* (4), 1952–1961. https://doi.org/10.1021/acscatal.0c05599.

(6) Wang, Y.; Qin, R.; Wang, Y.; Ren, J.; Zhou, W.; Li, L.; Ming, J.; Zhang, W.; Fu, G.; Zheng, N. Chemoselective Hydrogenation of Nitroaromatics at the Nanoscale Iron(III)–OH–Platinum Interface. *Angew. Chem. Int. Ed.* **2020**, *59* (31), 12736–12740. https://doi.org/10.1002/anie.202003651.

(7) Trandafir, M. M.; Neaţu, F.; Chirica, I. M.; Neaţu, Ş.; Kuncser, A. C.; Cucolea, E. I.; Natu, V.; Barsoum, M. W.; Florea, M. 7-Highly Efficient Ultralow Pd Loading Supported on MAX Phases for Chemoselective Hydrogenation. *ACS Catal.* **2020**, *10* (10), 5899–5908. https://doi.org/10.1021/acscatal.0c00082.

(8) Lin, L.; Yao, S.; Gao, R.; Liang, X.; Yu, Q.; Deng, Y.; Liu, J.; Peng, M.; Jiang, Z.; Li, S.; Li, Y.-W.; Wen, X.-D.; Zhou, W.; Ma, D. 8-A Highly CO-Tolerant Atomically Dispersed Pt Catalyst for Chemoselective Hydrogenation. *Nat. Nanotechnol.* **2019**, *14* (4), 354–361. https://doi.org/10.1038/s41565-019-0366-5.
